# Food hygiene practices and associated factors among food handlers in food establishments of Jimma City, Southwest Ethiopia

**DOI:** 10.1371/journal.pone.0321222

**Published:** 2025-05-29

**Authors:** Megersa Werku Tesso, Mulunesh Deti, Temima Jemal, Dessalegn Dadi, Abera Gure, Derartu Nasir, Dechasa Befikadu, Habtamu Roba

**Affiliations:** 1 Department of Public Health, Institute of Health, Dambi Dollo University: Dambi Dollo, Ethiopia; 2 Department of Environmental Health Science and Technology, Faculty of Public Health, Institute of Health, Jimma University, Jimma, Ethiopia; 3 Department of Chemistry, College of Natural Sciences, Jimma University, Jimma, Ethiopia; 4 Environmental Protection Office, Jimma Zone, Jimma, Oromia, Ethiopia; 5 Department of Medical Laboratory Science, Institute of Health, Dambi Dollo University, Dambi Dollo, Ethiopia; 6 Department of Environmental Health Science, Institute of Health, Bule Hora University, Bule Hora, Ethiopia; BHU: Bule Hora University, Institute of Health, ETHIOPIA

## Abstract

**Background:**

Food hygiene has been a global headache due to its high impact on human health as well as its adverse effects on socioeconomic productivity. It is important to recognize the worth of food handlers’ hygienic practices at food establishments to achieve community health. However, little is known about food handlers’ food hygienic practice and its associated factors with the huge increment of food establishments in the study area.

**Objective:**

This study aimed to assess food hygiene practices and associated factors among food handlers working in food establishments in Jimma City, Southwest Ethiopia.

**Methods:**

An institution-based cross-sectional study was conducted using a simple random sampling technique to select a total of 316 food handlers by using a semi-structured questionnaire. After the interviews, hand swabs were collected and immediately transferred to the microbiology lab for the isolation of selected bacteria. The swab samples were cultured and identified using standard operating procedures. The data were entered into Epi Data (version 4.6.0) and exported to SPSS (version 29) for further analysis. Binary logistic regression was then used, with a p-value of 0.05 considered as an associated factor.

**Results:**

The study results showed that only 28.4% of the food handlers had good food hygiene practices. Additionally, 45.1% of the 310 swabs collected from the hands of food handlers tested positive for bacterial contamination, with *Staphylococcus aureus* being the most predominantly isolated bacteria. Role as waiter (AOR = 0.48, 95% CI: 0.23, 0.99), the type of food establishment (bar and restaurant) (AOR = 0.37, 95% CI: 0.15, 0.88), being negative for isolated bacteria (AOR = 2.95, 95% CI: 1.42, 6.13), having a positive attitude (AOR = 2.32, 95% CI: 1.13, 4.73), and having good knowledge (AOR = 8.23, 95% CI: 4.03, 16.78) toward food hygiene were among associated factors of food hygiene practices.

**Conclusion:**

The majority of food handlers were found to have poor food hygiene practices, indicating low adherence to proper food handling practices. Emphasis should be placed on delivering periodic and effective health education to improve food handlers’ hygiene practices.

## Introduction

Food hygiene is an essential condition and measure to endorse food safety from farm to fork, which principally prevents microbial contamination of food at all points [1]. Health and productivity are enhanced by the provision of hygiene food, which creates an efficient platform for development and poverty alleviation [2]. Food hygiene became the global burning issue due to its potential role in public health and the need to mitigate foodborne diseases (FBDs) caused by unsafe food handling practices [3]. Unsafe food practices continue to be a major cause of morbidity and mortality around the world, with billions of people at risk of consuming contaminated food [4].

According to WHO statistics of 30 April 2020 and Zanin et al., it is estimated that nearly one out of every ten people was ill as a result of consuming contaminated food items, resulting in 600 million people becoming ill each year and approximately 420,000 of these victims dying each year [5]. FBDs are common in developed and developing countries due to poor food hygiene and sanitation practices [1,6]. However, Africa, Southeast Asia, and the Eastern Mediterranean are thought to have the highest incidence and death rates from FBDs. Additionally, WHO reported that contaminated food causes 1.5 billion cases of diarrhea in children each year, resulting in more than three million premature deaths [7]. Yet, it is expected that many illnesses remain underreported, as only the most severe cases are usually investigated in most developing countries because reliable statistics on foodborne diseases are unavailable because of poor reporting systems [4]. Lack of safe water, inappropriate food storage facilities, and poorly enforced food safety laws contribute to a higher prevalence of FBIs in low-income countries and food establishments [8,9].

A meta-analytic study conducted in the USA and Ethiopia showed that many of these FBDs emerged from inadequately handled food in food service establishments [10,11]. The cross-contamination of cooked and raw food, uncleaned food contact surfaces, and poor personal hygiene are other contributing factors of poor food hygiene that cause FBD outbreaks [12,13]. Another meta-analytical study illustrated that a lack of food safety training, regular medical checkups, hand washing facilities, and formal education were among the factors contributing to the low prevalence of food hygiene practices among Ethiopian food handlers [14]. Similarly, a lack of catering facilities, such as a functional handwashing facility and a liquid and solid waste disposal facility, and a food hygiene awareness were associated with food hygiene practices [15,16]. Additionally, poor personal hygiene, which is strongly linked to poor food hygiene practices, maybe a source of infections and a reservoir for antimicrobial resistance genes [17].

Moreover, in addition to guarding food security, food hygiene is also a public health action area to protect consumers from food poisoning and FBD since unsafe foods can contain pathogenic bacteria. *Staphylococcus aureus, Escherichia coli, Shigella, Salmonella,* and *Pseudomonas* are among the most predominant antibiotic-resistant bacteria that cause food contamination and negatively affect food handlers’ food hygiene in food establishments [18–20]. Food handlers who do not follow safe food handling practices can potentially harbor and transmit pathogenic bacteria that can contaminate food items, ultimately affecting the health of consumers [21]. A previous study conducted on bacterial hand contamination among food handlers working in various canteens in India found that the bacteria isolated from the food handlers’ hands were highly associated with their hand washing habits [22]. So, it is crucial to follow hand hygiene instructions as part of food hygiene to prevent and control FBIs linked to food serving catering [23]

Globally, the number of food establishments has expanded due to progressive urbanization, industrialization, and tourism growth, which might compromise food hygiene unless strict food hygiene practices were followed [24]. Jimma City is a rapidly growing urban center in Ethiopia, which is not unique in experiencing a surge in the number of food establishments. Despite this significant increase in food establishments in the city, there is a lack of information on the food hygiene practices of food handlers working in these facilities. Food handlers play a critical role as the last line of defense against FBDs because they are responsible for ensuring food hygiene and safety within food establishments [25]. The previous study in this study setting was conducted among food handlers who worked in university student cafeterias, which have better infrastructure than the private food establishments that are the focus of the current study. Additionally, most of previous studies on food handlers’ hygiene practices did not assess bacterial hand contamination as a contributing factor, which is a key focus of this study. Therefore, the aim of this study is to assess the food hygiene practices and associated factors among food handlers working in food establishments in Jimma City. The study findings will inform appropriate interventions to address foodborne diseases (FBDs) by identifying factors linked to food hygiene practices. This study can also serve as a basis for further research in this field.

## Methods and materials

### Study setting

**Fig 1:**
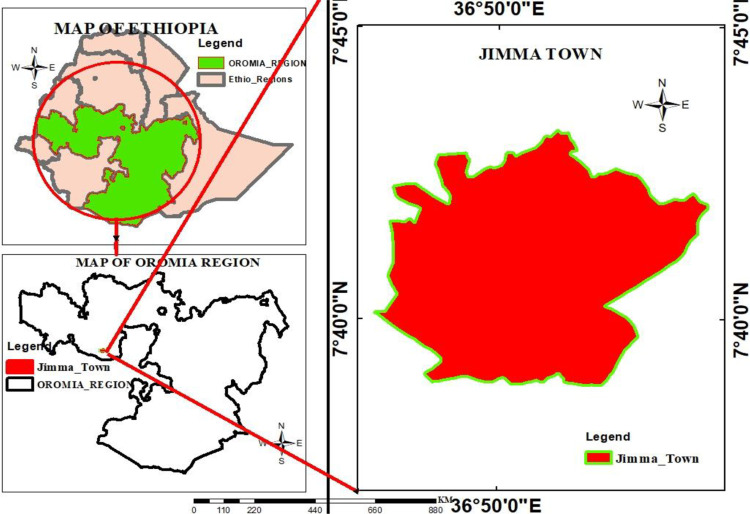
Map of the study area, Jimma City, Southwest Ethiopia, 2023.

The study was conducted in Jimma City, Southwestern Ethiopia, 356 kilometers from Addis Ababa, Ethiopia’s capital city. The city is situated at latitudes ranging from 7° 40′ N to 36° 50′ E and elevations ranging from 880 to 3360 meters above sea level. Based on the Central Statistical Agency (CSA, 2021), Jimma has a population of 258,570, and grows by 4.9% annually. The Jimma city has 17 smalls administrative kebeles (Fig 1). There is a warm and humid climate in the study area, and the mean annual rainfall is about 1530 mm [26]. According to Jimma city Cultural and Tourism bureau, about 250 food establishments (66 hotels, 79 bar and restaurants, 50 café and restaurants and 55 snack houses) were registered in Jimma city.

### Study design

An institution-based, cross-sectional study was implemented to assess food hygienic practices and associated factors among food handlers working in food establishments in Jimma city.

### Study period

This study was carried out from 18 May to 24 June 2023 among food handlers working in food establishments in Jimma city.

### Source and study population

All food handlers who were working in food establishments in Jimma city where source population whereas food handlers who had the frequent contact with food and participates in food handling operations and who were available during the data collection period are participants of the study. Food handlers who were unable to communicate due to a disability and who had skin irritation or eczema on their hands were excluded from the study. Furthermore, food handlers who had a disease and being on the treatment were excluded.

### Sample size and sampling procedure

#### Sample size.

The sample size was calculated using a single population proportion formula and the assumption that the probability of food hygiene practices among food handlers is 27.4%, as reported by a similar previous study in the Bole sub-city, Addis Ababa, Ethiopia [27] and by considering other assumptions: 95% confidence interval (CI) and 5% margin of error

n=(Za/2)2*P(1−p)d2; n=[1.96] ^2x0.274x0.726[0.05] ^2=301

Where n= the sample size, P = proportion of food hygiene practices, and z = z-statistics value that corresponds to the 95 percent cut-off point in the standard normal distribution, d = marginal error. By considering a 5% non-response rate, the total sample size was 316.

### Sampling procedure

**Fig 2:**
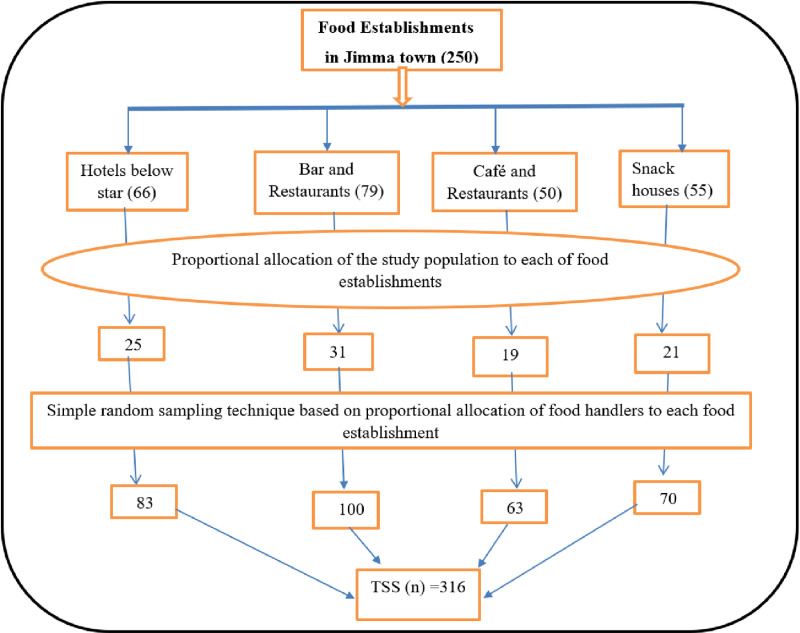
Schematic diagram of the sampling frame for food hygiene practices among food handlers working in food establishments, in Jimma City, Southwest Ethiopia, 2023.

According to the Jimma City Cultural and Tourism Bureau, about 250 food establishments (66 hotels, 79 bars and restaurants, 50 cafes and restaurants, and 55 snack houses) were registered in Jimma City. The bureau faced challenges in determining the total number of food handlers employed in the food establishments due to the lack of relevant recorded documents. The study population was proportionally allocated to each food establishment to obtain a representative sample size. Then, after a list of all food establishments was obtained from this bureau, a simple random sampling technique was used. Then, a current list of food handlers was obtained from the manager or owner of the selected food establishment, and a proportional allocation was made based on the number of food handlers. Finally, a food handler was randomly chosen by using a lottery method from the cooker and waiter in the presence of one or more other food handlers, using their roster as a sampling frame. As a result, 83 food handlers from the hotels, 100 food handlers from bar and restaurants, 63 food handlers from café and restaurants, and 70 food handlers from snack houses were selected (Fig 2).

### Study variables

#### Dependent variable.

The outcome variable of this study was food hygiene practices, which is taken as binary (“1” good food hygiene practices or “0” poor food hygiene practices) based on food handlers practice on food hygiene.

### Independent variable

Socio-demographic factors (sex, age, marital status, educational status and year of service), working position, type of food establishments, knowledge of food hygiene practices, attitude towards food hygiene practices and bacterial hand contamination of food handlers.

### Operational definitions

#### Food establishments.

Establishments that engaged in providing food and drinking services to virtually large groups of people. These establishments include hotels, bars, restaurants, cafeterias, and snack houses [28].

#### Food handlers.

anyone who work in food establishments and who serve/prepare food or who had frequent contact with a food material.

#### Food hygiene practices.

a process that food handlers carried out to protect food from contamination and make sure it’s safe for consumers [29].

For the purpose of dichotomous scores from hygiene practice, attitude and knowledge were converted to percentage and 70% were used as cutoff point.

#### Food hygiene practice.

Food handlers who had scored less than or equal to 70% of their responses to the total food hygiene practice-related questions were considered to have a poor level of practice. Those who scored more than 70% were considered to have a good level of practice. In other words, food hygiene-related questions were measured with a five-point Likert scale that was reversed for some negative statements/questions to check the validity of the respondents’ responses. The range of possible scores is 8–40, with higher scores above 30 (more than 70%) representing a good food hygiene practice.

#### Food hygiene knowledge.

Food handlers who had scored less than or equal to 70% of their responses to the total food hygiene knowledge-related questions were considered to have a poor level of knowledge. Those who scored more than 70% were considered to have a good level of knowledge.

#### Food hygiene attitude.

Food handlers who had scored less than or equal to 70% of their responses to the total food hygiene attitude-related questions were considered to have a negative level of attitude. Those who scored more than 70% were considered to have a positive level of attitude.

#### Bacterial isolates.

are types of strains identified through a series of procedures and investigations in the laboratory from hand swab samples containing a mixed population of bacteria that may have the potential to cause food-borne disease [30,31].

### Data collection techniques and procedures

#### Data collection.

The data on food hygiene practices and associated factors was collected by face-to-face interviews using pre-tested semi-structured questionnaires. A Questionnaire was developed from WHO [32] guidelines on safe food handling and after reviewing relevant literature [27,33–36]. The questionnaire consists of four parts, which include a total of 31 items: 7 questions on the socio-demographic characteristics of food handlers, 8 food hygiene practice-related questions, 9 food hygiene knowledge-related items, and 7 items on food handler’s attitude toward food hygiene practice. “S2 Annex II” After the interview, participants were asked to provide hand rinse samples in a sterile test tube for bacterial analysis.

### Laboratory procedures

Before/after starting any meal preparation activity, after hand washing as usual (if any), the participant’s hands were sampled for bacterial testing. There was no advance notice, and extra hand hygiene was not permitted during the collection of hand rinse samples. To collect samples, sterile cotton swabs (Puritan low-lint cylindrical tip) were prepared and dipped into sterile test tubes containing saline. Samples from each participant’s hand contents were collected from all over the hand by rubbing cotton swabs in a moistened state [37]. Swabs were collected with the help of a swab stick, which had sterile cotton buds soaked in 0.85% of 5 ml of saline solution. Thereafter, the tubes were labeled and put into a cold box (<4°C) and transported to the Microbiology laboratory at Jimma University for analysis within 4 hours of collection [38].

**Fig 3:**
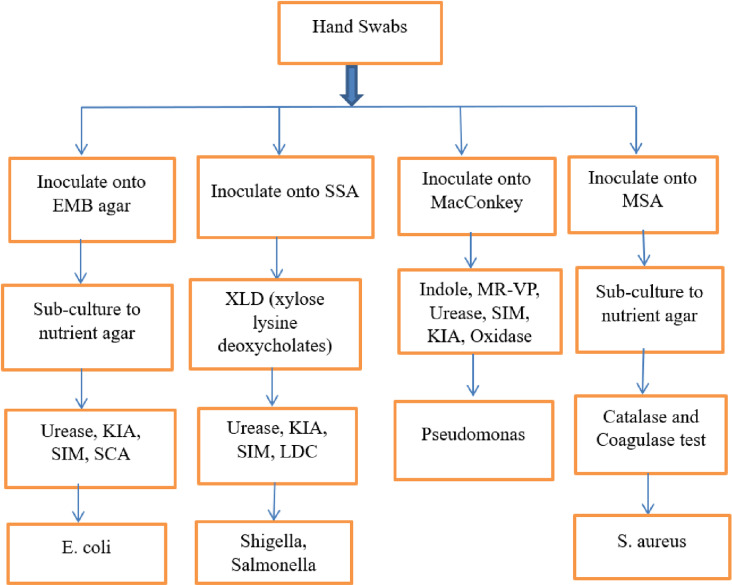
Laboratory flowchart for bacterial isolation from food handlers’ hands working in food establishments, in Jimma City, Southwest Ethiopia, 2023.

Then, for the confinement of S. aureus, swabs obtained from the hands of participants were refined quickly on mannitol salt agar (MSA). Whereas for the confinement of gram-negative microscopic organisms, swabs obtained from the hands of participants were refined onto MacConkey agar (Difco), Salmonella-Shigella agar (SSA) (Oxoid), and after that was incubated at 37°C for 24 hours. The colony of microscopic organisms developed on the media was recognized by the morphology of its colonial and a battery of biochemical tests like a response on catalase, oxidase, Simmon citrate, urease, indole generation, Kliger Iron agar, motility, and gas and hydrogen sulfide (H2S) generation [39–41]. Accordingly, the general laboratory flow chart for bacterial isolate was amended as shown in (Fig 3).

### Data quality management

Questionnaires were translated into a local language (i.e., Amharic and Afan Oromo) for data collection and retranslated back into English upon data collection completion to ensure data quality. Before the actual data collection, the questionnaire was designed correctly and pre-tested in the nearby town of Agaro in the Jimma zone by taking 5% of the total sample size of study participants to ensure the clarity and reliability of the survey tools. The necessary correction was made to ensure completeness before data gathering and entry. The accuracy of a sample of completed data was verified, and data was entered twice and compared. Furthermore, it was checked for outliers, and proper handling of missed values was executed.

The data collectors received a two-day training on the study’s objective, significance, information confidentiality, respondent’s rights, and interview techniques. Four environmental health officers and a microbiologist were involved in data collection and laboratory tasks respectively, with an additional MSc in environmental health serving as a supervisor. Throughout the data collection process, the principal investigator and supervisor closely monitored the process, ensuring completeness and providing intensive supervision. During the laboratory analysis, SOP was strictly followed to ensure the quality of the laboratory’s analysis. All instruments used were routinely inspected for proper functionality, and quality control was maintained using reference strains of *S. aureus (ATCC 6538) and E. coli (ATCC 25922)* that were obtained from Jimma University microbiology laboratory. Subsequently, 5% of the batch was incubated at 35–37 °C overnight, and bacterial growth was monitored to ensure the culture media were sterile. Any media that depicts any microorganism’s growth was discarded.

### Ethical approval and Informed consent

Ethical clearance was obtained from the Institutional Review Board of Jimma University Institute of Health with Ref. No: JUIH/IRB/354/2023. Following approval letter, a written official letter of cooperation was given to the Jimma city cultural and tourism bureau and corresponding food establishments. Informed written consent was obtained from all participants after explanation of the purpose of the study and procedure of sample collection. Then, respondents were described to and guaranteed that the data collected was treated with utmost privacy, hence it was the duty of the investigator. Participation was fully voluntary and we explained them that they could withdraw participation and asking any question not clear at any time of the interview were the rights they have. **“**S1 Annex I**”**

### Data entry and analysis

The collected data was compiled, checked for any inconsistencies and missed values, coded, and entered using Epi-data version 4.6.0 software, then exported into SPSS Statistics (Version 29.0.1.0) and for data analysis. By running frequencies and crosstabs, missing values were cleaned from the data. Cronbach’s alpha was used to assess the tools’ reliability which was 0.76 for the outcome variable. Descriptive analysis was conducted to describe the frequencies, percentages, mean and standard deviation. To evaluate the degree of relationship between independent and dependent variables, an odds ratio with a 95% CI was used. At a p-value of less than 0.05, an association was declared statistically significant. The adjusted model’s fitness was assessed using the Hosmer and Lemeshow goodness-of-fit test. The findings were presented in text as a percentage, odds ratio (OR), tables, and charts. “S3 Supplementary Data**”**

## Results

### Socio-demographic characteristics of food handlers

A total of 310 food handlers participated in this study, with a response rate of 98.1%. Almost three-fourths (73.9%) of the participants were female. The mean age of the participants was 23.8 years (SD = 6.12), and nearly half (49.7%) were between the ages of 20 and 30. Approximately two-thirds (64.5%) of the participants were single in terms of their marital status, and only 16.2% had attended high school or above. In terms of food handlers’ experience, more than half (61.3%) of the respondents had worked for less than two years (Table 1).

**Table 1:** Food handlers’ socio-demographic characteristics in food establishments in Jimma City, Southwest Ethiopia, 2023 (n=310).

Characteristics	Category	Frequency(N)	Percent (%)
Sex	Male	81	26.1
	Female	229	73.9
Age	<= 20	115	37.1
	21–30	154	49.7
	31–40	34	11.0
	> 40	7	2.3
Marital Status	Single	200	64.5
	Married	98	31.6
	Divorced	11	3.5
	Widowed	1	.3
Educational Status	Can’t read and write	11	3.5
	Primary education	119	38.4
	Secondary education	130	41.9
	Diploma and above	50	16.1
Year of Service	< 2	190	61.3
	2–4	80	25.8
	5–7	29	9.4
	> = 8	11	3.5
Role	Cooker	160	51.6
	Waiter	150	48.4
Food Establishment	Hotel	82	26.5
	Bar and Restaurant	98	31.6
	Cafeteria and Restaurant	62	20.0
	Snack House	68	21.9

### Knowledge of food handlers towards food hygiene

Most of the study participants (87.7%) knew that germs could grow on unclean cutting boards. In contrast, one third of them (33.9%) knew that the health status of workers should be evaluated before employment. Moreover, the overall knowledge of food hygiene practices among food handlers was found to be 31.9% (Table 2).

**Table 2:** Knowledge of food handlers toward food hygiene practices in food establishments in Jimma City, Southwest Ethiopia, 2023 (n=310).

Knowledge related characteristics	Response	
	Yes	No
Know about food borne disease transmission due to contaminated food	191(61.6)	119(38.4)
Improper handling of food could pose health risks to consumers	279(90.0)	31(10.0)
Know food prepared in advance reduces the risk of food contamination	269(86.8)	41(13.2)
Know germs can be found on cutting board	272(87.7)	38(12.3)
Contaminated foods always have some change in color, odor or taste	203(65.5)	107(34.5)
Know health status of workers should be evaluated before employment	105(33.9)	205(66.1)
Know use of safe water for cooking purpose can reduces FBDs	190(61.3)	120(38.7)
Know vectors (like fly, rats and cockroaches) lead for food contamination	286(92.3)	24(7.7)
Know hand drying is as important as to hand washing during food handling	239(77.1)	71(22.9)

**#**Numbers in the parentheses shows corresponding percentage

### Attitude of food handlers towards food hygiene practice

Less than one-third (28.7%) of participants strongly agreed that long and painted fingernails could contaminate food with foodborne pathogens. Additionally, nearly half (49.0%) of food handlers agreed that safe food handling is an essential part of their job responsibilities. However, overall, only 33.5% of food handlers had a positive attitude toward practicing food hygiene (Table 3).

**Table 3:** Attitude of food handlers toward food hygiene practice in food establishments in Jimma City, Southwest Ethiopia, 2023 (n=310).

Attitude Related Characteristics	Response				
	Strongly agree	Agree	Undecided	Disagree	Strongly Disagree
Improper food storage is dangerous to health	50(16.1)	180(58.1)	62(20.0)	18(5.8)	0(0.0)
Frequent hand-washing during food preparation is worth the extra time	63(20.3)	136(43.9)	82(26.5)	28(9)	1(0.3)
Keeping working surfaces and utensils clean reduces the risk of illness	85(27.4)	193(62.3)	27(8.7)	4(1.3)	1(0.3)
Keeping raw and cooked food separate prevent illness	89(28.7)	159(51.3)	41(13.2)	20(6.5)	1(0.3)
Safe food handling is an important part of your job responsibilities	64(20.6)	152(49.0)	72(23.2)	20(6.5)	2(0.6)
Important to throw away foods that have reached their expiry date	137(44.2)	129(41.6)	28(9)	15(4,84,8)	1(0.3)
Long and painted fingernails could contaminate food with foodborne pathogens	89(28.7)	118(38.1)	54(17.4)	48(15.5)	1(0.3)

### Food hygiene practice of food handlers

Only nearly one-third (30.3%) of food handlers use gloves when handling ready-to-eat food or preparing food. Additionally, more than half (60.0%) of the respondents admitted to attending their routine work when they had a cold (Table 4).

**Table 4:** Food hygiene practices among food handlers working in food establishments in Jimma City, Southwest Ethiopia, 2023 (n=310).

Hygiene practice related characteristics	Response				
	Always	Most of the times	Sometimes	Rarely	Never
Always use clean and separate cutting boards for raw and ready-to-eat food	142(45.8)	126(40.6)	19(6.1)	23(7.4)	0(0.0)
I don’t work when I have diarrhea	4(1.3)	102(32.9)	97(31.3)	101(32.6)	6(1.9)
Use water storage equipment in this food establishment	106(34.2)	136(43.9)	46(14.8)	18(5.8)	4(1.3)
I don’t wear any kind of bracelets during food serve/preparation	33(10.6)	137(44.2)	58(18.7)	76(24.5)	6(1.9)
I don’t keep cooked meal at room temperature for more than 4 hours	19(6.1)	152(49)	74(23.9)	59(19)	6(1.9)
Wear gloves when you handle ready to eat food	12(3.9)	94(30.3)	112(36.1)	83(26.8)	9(2.9)
Always wash your hand with water and soap after using the toilet	97(31.3)	182(58.4)	26(7.7)	5(1.6)	0(0.0)
I don’t work when I have cold	3(1.0)	38(12.3)	69(22.3)	186(60)	14(4.5)

### Level of food hygiene practice

**Fig 4:**
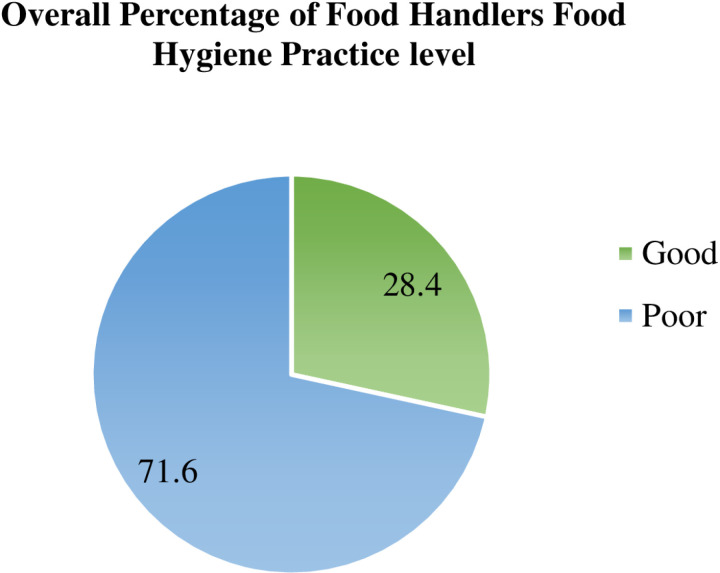
Overall food hygiene practices of food handlers working in food establishments in Jimma City, Southwest Ethiopia, 2023 (n=310).

Overall, among all respondents, less than one-third (28.4%), with a 95% CI of [23,33] of study participants had good food hygiene practices. In contrast, nearly three-quarters (71.6%) of them demonstrated poor food hygiene practices (Fig 4).

### Isolation of bacteria on hand of food handlers

**Fig 5:**
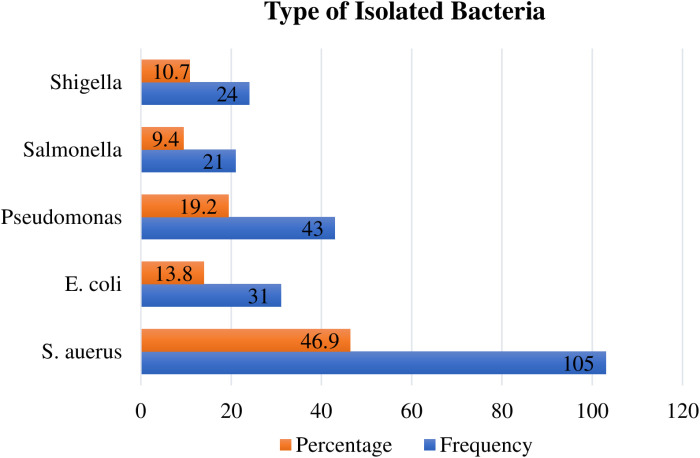
Types of bacteria Isolated from hand swap samples of food handlers working in Food Establishments in Jimma City, Southwest Ethiopia, 2023 (n=310).

A total of 310 food handlers’ hand swab samples were taken and examined for the presence of selected bacteria. Nearly half (45.2%) of the study participants tested positive for one or more bacterial hand contaminants. A total of 224 bacteria were identified on the hands of the study participants, with *S. aureus* being the most predominantly isolated. However, bacteria were not isolated from the hand swabs of more than half (54.8%) of the study participants (Fig 5).

### Isolated bacteria and its associated factors

There was a statistically significant correlation between the frequency of isolated bacteria and the experience of a food handler at a P-value of < 0.05. Accordingly, the bacterial isolation from the hands of food handlers showed significant associations with their work experiences as food handlers (X2 = 68.47, DF = 3, P = 0.00). The isolation rate of bacterial hand contaminant was lower (17.5%, 10.3%, and 18.2% among food handlers who served for 2–4, 5–7, and above eight years, respectively, while it was higher (63.7%) among food handlers who had served for less than two years (Table 5).

**Table 5:** Isolated bacteria among food handlers and its associated factors in food establishments in Jimma City, southwest Ethiopia, 2023(n=310).

Variable	Presence of Isolated Bacteria		Pearson χ2 & asymptotic Sign (2 sided)
	Positive	Negative	
Work experience as food handlers			X^2^ (3) =68.465 P=.000
< 2	121(63.7)	69(36.3)	
2–4	14(17.5)	66(82.5)	
5–7	3(10.3)	26(89.7)	
> = 8	2(18.2)	9(81.8)	

#*Numbers in bracket shows percentage, P(P-value), X*^*2*^ *(Chi-square), DF= degree of freedom*

**Fig 6:**
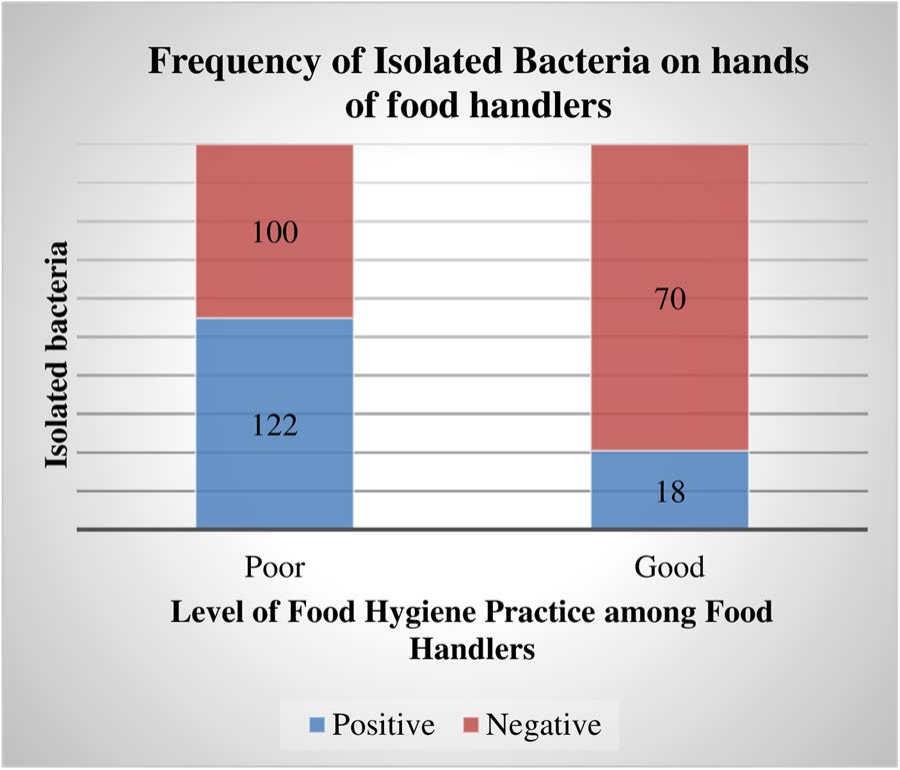
Bacteria Isolated from food handlers’ hands working in food establishments in Jimma City, Southwest Ethiopia, by food hygiene practice level, 2023 (n=310).

Moreover, there is a significant association between the level of food hygiene practices and the rate of bacterial isolation from the hands of food handlers (X2 [1] = 30.288, P = 0.000). The isolation rates of bacteria from hand swabs were relatively higher among the study participants, with 122 (87.1%) having poor levels of food hygiene practices compared to the 18 (12.5%) participants with good levels of hygiene practices (Fig 6). However, there is no significant relationship between bacterial isolation from food handlers’ hands and the participants’ role, sex, age, educational status, or marital status in the current study.

### Factors associated with food hygiene practices

The backward (LR) variable selection method was used in the multivariable logistic analysis to declare associated factors with food hygiene practices of food handlers working in food establishments in Jimma City (Table 6).

**Table 6:** Multivariable binary LR analysis and associated factors with food hygiene practice of food handlers working in food establishments in Jimma City, Southwest Ethiopia, 2023(n = 310).

Variables	Level of Food Hygiene practice		COR of 95% CI	AOR of 95% CI
	Poor	Good		
Role of participants				
Cooker	101	59	1	1
Waiter	121	29	0.434(0.262, 0.718) *	0.48(0.230,0.989) *
Age of participants				
20 and under	99	16	1	1
Above 20	123	72	3.622(1.982, 6.619) **	≠
Experience(year) of participant				
<2	159	31	1	1
2-4	44	36	4.196(2.338, 7.532) **	≠
5-7	12	17	7.266(3.158, 16.716) **	
>=8	7	4	2.931(0.809, 10.619)	
Educational status				
Can’t read and write	11	4	1	1
Primary education	94	23	0.673(0.196, 2.306)	≠
Secondary education	94	34	0.995(0.297, 3.335)	
Diploma and above	23	27	3.228(0.904, 11.522)	
Type of food establishments				
Hotel	39	43	1	1
Bar and Restaurant	76	22	0.240(0.122,0.471) **	0.37(0.152,0.881) *
Cafeteria and Restaurant	48	14	0.247(0.121,0.506) **	0.35(0.138,0.884) *
Snack House	59	9	0.123(0.055,0.277) **	0.16(0.057,0.436) **
Presence of isolated bacteria				
Positive	122	18	1	1
Negative	100	70	4.744(2.652, 8.487) **	2.95(1.420,6.134) **
Level of attitude				
Negative	175	31	1	1
Positive	47	57	6.85(3.977,11.785) **	2.32(1.134,4.730) *
Level of knowledge				
Poor	187	24	1	1
Good	35	64	14.25(7.883, 25.752) **	8.23(4.026,16.778) **

NB: CI=Confidence Interval, 1 = reference category, [* = P-value (0.01–0.05), ** = p-value (<0.01)] (significant variables), ≠ = variables with p-value >0.05(insignificant) in multivariate binary logistic regression (backward LR).

Accordingly, the presence of isolated bacteria on the hands of food handlers was among significantly associated factors with food hygiene. Food handlers who tested negative for isolated bacteria were 2.95 times more likely to have good food hygiene practices than those who tested positive. This study also showed a significant association between working position (cooker or waiter) and food hygiene practices. Accordingly, food handlers who worked in service positions were 0.48 times less likely to have good food hygiene practices as compared to those who had been working as a cooker (AOR = 0.48, 95% CI: 0.230, 0.989).

Similarly, the odds of performing good food hygiene practices were about 84% less likely among food handlers who worked in snack houses as compared to food handlers who worked in hotels. In other words, food handlers who worked in snack houses were 0.16 times less likely to perform good food hygiene practices compared to food handlers who worked in hotels. Additionally, this study revealed that participants’ attitudes were significantly associated with good food hygiene practices. Food handlers who had positive attitudes were 2.32 times more likely to have good food hygiene practices than those who had negative attitudes toward food hygiene. Furthermore, food handlers who had good knowledge of food hygiene were 8.23 times more likely to have good food hygiene practices than their counterparts (AOR = 8.23, 95% CI: 4.026, 16.778) (Table 6).

## Discussion

Food hygiene has become a global issue of concern, particularly in developing countries like Ethiopia, due to its potential link to public health[3]. It is imperative to recognize food handler hygiene practices to ensure food hygiene, which, in turn, safeguards food security and community health. So, this study assessed food handlers’ hygienic practices and associated factors in food establishments in Jimma City.

This study illustrated that only 28.4% with a 95% CI of [23,33] of study participants had good food hygiene practices. This finding is in line with the survey conducted in Arba Minch town (32.6%) [42] and Addis Ababa Bole sub-city (27.4%)[27]. However, the finding of this study was lower than other studies conducted in Gondar (46.7%) [33], Debre Markos town (54%) [43], Egypt (56.3%) [44] and Indonesia (90%) [45]. This discrepancy may be due to study setting, food cultural differences, and a difference in the educational status of respondents. In this study, only 16.1% of food handlers attended high school and above. The study in Indonesia was conducted in healthcare facilities, and the survey in Gondar was done in the institution (university). Compared to the current study, these facilities might have sufficient infrastructure and appropriate management practices that may foster good hygienic practices [46]. It might also be linked to inequitable sanitary and premises conditions among food establishments, which can contribute to food handler’s poor adherence to food hygiene practices [47].

Similarly, our finding on food handlers hygiene practices was also lower than the studies conducted in Fiche town (50.5%) and Northwest Ethiopia (49.8%) [48]. This variation may be due to the difference in cutoff point, study period and a participant’s sociodemographic (year of service as a food handler). In our case, more than half (60%) of study participants served for less than two years, which could have contributed to these differences. Another reason could be study by Alamneh et al.[48] was conducted during the COVID-19 outbreak, which was in an era of escalation in awareness-raising campaigns on the promotion of handwashing, and the overall hygiene adopted had a significant impact on food hygiene [49].

Our study revealed that food handlers who work as waiters are less likely to practice good food hygiene than those who work as cookers. This finding is comparable to a study by Alamneh et al. [48] that showed a significant association between food hygiene practice and working position. Possible reasons for this difference include the fact that cookers are expected to have more knowledge and experience in food hygiene and that they work in a more controlled environment than waiters [44]. Additionally, our study found that food handlers who worked in hotels were more likely to practice good food hygiene than those who worked in other types of food establishments (bars and restaurants, restaurants and cafeterias, and snack houses). The possible reason might be that the hotel may provide a more conducive environment for workers that promotes their food hygiene practices [15]. Another possible explanation is that hotels may have more supportive management practices that encourage good food hygiene practices [50].

This study also found that food handlers with positive attitudes towards food hygiene were 2.32 times more likely to have good food hygiene practices than those with negative attitudes. This finding is consistent with previous studies conducted in Bole sub-city, Northwest Ethiopia, and a meta-analytic study, which all found a significant association between attitude and hygiene practice [10,27,51]. Food handlers’ attitudes substantially affect their food hygiene practices, and food handlers who have good attitudes are assumed to have good knowledge, which is the foundation of good food hygiene practices [44]. Food handlers’ level of food handling practice is influenced by their attitude, which is a proximal component in converting food hygiene into observable action [52].

Moreover, this study illustrated that food handlers with good knowledge of food hygiene were more likely to have good food hygiene practices than those with poor knowledge. The odds of performing good food hygiene practices were 8.23 times more likely among food handlers with good knowledge of food hygiene than those with poor knowledge. This finding is consistent with previous studies conducted in Debre Markos [43], Dangila town [53], and Gondar city[54] and Nigeria [55]. The possible explanation is that food handlers with a good understanding of food hygiene principles are more likely to follow and practice these principles in their daily tasks. However, this finding was inconsistent with the study conducted in Gondar [33] and the Addis Ababa, Yeka sub-city [56], which found no association between knowledge and food hygiene practices.

Furthermore, our study demonstrated a significant association between the presence of isolated bacteria and food hygiene practices. Accordingly, the likelihood of good food hygiene practices among food handlers who were negative for isolated bacteria was 2.95 times more likely than those who were positive for isolated bacteria. This finding was consistent with a study conducted in South Africa [57]. Food handlers are in close contact with food, and their hands are potential sources of contamination, which in turn affects food hygiene and causes food-borne illness [58,59]. Our finding implies a high probability of transmission of pathogenic bacteria from food handlers’ hands to food substances or equipment during meal preparation and serving.

In this study, *S. aureus* (46.9%) and *Salmonella* (9.4%) were among the highest and lowest isolated bacteria on the hands of food handlers, respectively. Regarding isolated *S. aureus*, our finding aligns with a previous study from Iran that reported S. aureus as the most (46%) common pathogen isolated from food handlers [30]. However, this result is higher than previous studies from the same study area(Jimma) that reported a lower rate of *S. aureus* [60] and Solapur city of India [22]. This variation could be because *S. aureus* is the exact pathogenic bacteria included in the inhabitant microflora of the skin, and nearly half of healthy people carry *S. aureus* in the anterior nostrils of the nose [61]. Another possible explanation could be the differences in the food handled/served since the community food establishments served various foods compared to those in institutions.

In the current study, the prevalence rate of *E. coli* in the hands of food handlers was 13.6%. This finding is comparable to the study conducted by Assefa et al. [62], which showed that the prevalence of *E. coli* is 10.9%. The isolation of *E. coli* displays the potential of fecal-oral transmission of pathogens, which indicates a lack of compliance with hand hygiene practices and a recent history of fecal contamination among food handlers [63].

The prevalence of *pseudomonas* was found to be 19.4% in our study. This finding is inconsistent with an earlier study in Iran, which reported a lower rate of *pseudomonas* from food handlers’ hands [30]. This disparity may be due to the type of food they serve since food items with high moisture contents (such as raw meat, dairy, fresh fruits, and vegetables) are the main potential source of *pseudomonas* unless strict food handling practices are followed [64]. Another reason could be *pseudomonas* has a wide range of temperature preferences for growth, and it can contaminate and spoil both ordinary and refrigerated food products, putting food handlers at risk [65].

Our findings from laboratory analysis also showed that among 310 food handlers who participated in the current study, 9.4% and 10.7% were positive for *salmonella* and *shigella*, respectively. This finding is higher than that of the study conducted by Assefa et al. [62] and Awol et al. [66]. This variation could be due to the study setting and differences in sociodemographic profile. Another reason is that the study by Assefa et al. was carried out on campus, and most of the study participants were well educated and trained as compared to the current study, which may positively affect their hand hygiene practice. Overall, bacteria were not isolated from the hand swabs of more than half (54.8%) of the study participants. A possible reason could be due to the fact that the food handlers practiced hand hygiene (rubbing with alcohol or hand washing with soap and water) before a test was conducted, which can reduce bacterial hand contamination [67]. However, this study was not without limitations. One of the findings of this study is that it investigated self-reported food hygiene practices. Respondents may have been hesitant to provide answers that reflect negatively on themselves or their food establishment, which could have introduced bias. Additionally, due to resource constraints, *G. lamblia and E. histolytica* were not studied and an antimicrobial susceptibility test was not conducted for the isolated bacteria.

## Conclusion

The findings of this study clearly revealed that majority of food handlers had poor food hygiene practices. Nearly half of the study participants tested positive for pathogenic bacteria on their hands, which indicated that they did not adhere to proper food handling practices. Good knowledge, positive attitudes, work in cooking positions, working in hotels and negative for potential foodborne bacteria on the hands of food handlers were among the associated factors of good food hygiene practices. Emphasis should be placed on good food hygiene practices by enhancing food handlers’ knowledge and attitudes through training, as this is a critical method to achieving compliance with food hygiene practices. Jimma Cultural and Tourism must ensure compliance of food hygiene practices as the first criterion before issuing work permits for food establishments.

## Supporting information

S1 Annex I English version of the participant Information Sheet form. (DOCX)

S2 Annex II English Version of the participant Voluntary Consent form and Questionnaire. (DOCX)

S3 Supplementary Data Datasets of Analyzed Result in Tables form. (DOCX)

## References

[1] Kamboj S, Gupta N, Bandral J, Gandotra G, Anjum N. Food safety and hygiene: A review. Int J Chem Stud. 2020 Mar 1. 8:358–68.

[2] Kroger M.Comprehensive Reviews in Food Science and Food SafetyImpact Factor: 5.974ISI Journal Citation Reports. Journal of Food Science Education of Food Science. 2016

[3] Dagne H, Raju RP, Andualem Z, Hagos T, Addis K. Food Safety Practice and Its Associated Factors among Mothers in Debarq Town, Northwest Ethiopia: Community-Based Cross-Sectional Study. BioMed Res Int. 2019 Jun 4. 2019:1–8. 10.1155/2019/1549131 PMC6582849 31275961

[4] Fung F, Wang H-S, Menon S. Food safety in the 21st century. Biomed J. 2018 Apr;41(2):88–95. doi: 10.1016/j.bj.2018.03.003 29866604 PMC6138766

[5] Zanin LM, da Cunha DT, de Rosso VV, Capriles VD, Stedefeldt E. Knowledge, attitudes and practices of food handlers in food safety: An integrative review. Food Res Int. 2017 Oct;100:53–62. doi: 10.1016/j.foodres.2017.07.042 28873718

[6] Ansari MA, Khan Z. An Evaluation of Health Education Intervention on Hygienic Status of Food Handlers in Aligarh - A Three Year Experience. J Community Med Health Educ. 2012 Jan 1; 02.

[7] WHO. WHO surveillance programme for control of foodborne infections and intoxications: 8th report. J Infect. 2016;43(1):80–4. 10.1053/jinf.2001.0861 11597167

[8] Alemnew B, Gedefaw G, Diress G, Bizuneh AD. Prevalence and factors associated with intestinal parasitic infections among food handlers working at higher public University student’s cafeterias and public food establishments in Ethiopia: a systematic review and meta-analysis. BMC Infect Dis. 2020;20(1):156. doi: 10.1186/s12879-020-4884-4 32075585 PMC7031974

[9] WHOWHO estimates of the global burden of foodborne diseases: foodborne diseases burden epidemiology reference group 2007-2015. 2015.

[10] Tamene A, Habte A, Handiso D, Afework A, Endale F, Gizachew A, et al. Food safety practice and associated factors in public food establishments of Ethiopia: A systematic review and meta-analysis. PLOS ONE. 2022 May 27; 17: e0268918. 10.1371/journal.pone.0268918 PMC9140249 35622837

[11] Viator K, Blitstein J, Brophy J, Fraser A. Preventing and Controlling Foodborne Disease in Commercial and Institutional Food Service Settings: A Systematic Review of Published Intervention Studies. J Food Prot. 2015 Feb 1; 78:446–56. 25710165 10.4315/0362-028X.JFP-14-266

[12] Odeyemi OA, Sani NA. Antibiotic resistance and burden of foodborne diseases in developing countries. Future Sci OA. 2016 Dec;2(4):FSO 139. 10.4155/fsoa-2016-0023 PMC5242143 28116122

[13] Nahar N, Mahyudin NA. Microbiological Quality of Food Contact Surfaces (Spoons) at Selected Restaurants in Klang Valley, Malaysia. Sains Malays. 636685920000000000;47(07):1541–5.

[14] Negassa B, Ashuro Z, Soboksa NE. Hygienic Food Handling Practices and Associated Factors Among Food Handlers in Ethiopia: A Systematic Review and Meta-Analysis. Environ Health Insights. 2022 Jun 4; 16:11786302221105320. 35677751 10.1177/11786302221105320 PMC9168867

[15] Okugn A, Woldeyohannes D. Food hygiene practices and its associated factors among model and non-model households in Abobo district, southwestern Ethiopia: Comparative cross-sectional study. PLoS One. 2018;13(4):e0194391. doi: 10.1371/journal.pone.0194391 29621267 PMC5886398

[16] Kamboj S, Gupta N, Bandral JD, Gandotra G, Anjum N. Food safety and hygiene: A review. Int J Chem Stud. 2020;8(2):358–68.

[17] Mashak Z. Prevalence and Antibiotic Resistance of *Escherichia coli* O157:H7 Isolated from Raw Meat Samples of Ruminants and Poultry. J Food Nutr Res. 2018 Feb 6;6(2):96–102.

[18] Eltai NO, Yassine HM, Al Thani AA, Abu Madi MA, Ismail A, Ibrahim E, et al. Prevalence of antibiotic resistant Escherichia coli isolates from fecal samples of food handlers in Qatar. Antimicrob Resist Infect Control. 2018;7:78. doi: 10.1186/s13756-018-0369-2 29983931 PMC6019201

[19] Wakabayashi Y, Umeda K, Yonogi S, Nakamura H, Yamamoto K, Kumeda Y, et al. Staphylococcal food poisoning caused by Staphylococcus argenteus harboring staphylococcal enterotoxin genes. Int J Food Microbiol. 2018 Jan 16; 265:23–9. 29112896 10.1016/j.ijfoodmicro.2017.10.022

[20] Vicar EK, Alo DB, Koyiri VC, Opare-Asamoah K, Obeng-Bempong M, Mensah GI. Carriage of Antibiotic Resistant Bacteria and Associated Factors Among Food Handlers in Tamale Metropolis, Ghana: Implications for Food Safety. Microbiol Insights. 2023;16:11786361221150695. doi: 10.1177/11786361221150695 36726578 PMC9885032

[21] Alem K. Bacterial Load Assessment of some Food Items Sold in Street in Woldia Town, North-East Ethiopia. J Pure Appl Microbiol. 2020 Sep 30;14(3):1845–54.

[22] Sharma A, Subarnolata G, Jugal Kishor A, Amit KV. Ingole2. Hand Contamination among Food Handlers: A Study on the Assessment of Food Handlers in Canteen of Various Hospitals in Solapur City, Maharashtra. Journal of Pure and Applied Microbiology. 2021.

[23] Wiedenmayer K, Msamba V-S, Chilunda F, Kiologwe JC, Seni J. Impact of hand hygiene intervention: a comparative study in health care facilities in Dodoma region, Tanzania using WHO methodology. Antimicrob Resist Infect Control. 2020;9(1):80. doi: 10.1186/s13756-020-00743-4 32513311 PMC7282123

[24] Akabanda F, Hlortsi EH, Owusu-Kwarteng J. Food safety knowledge, attitudes and practices of institutional food-handlers in Ghana. BMC Public Health. 2017 Jan 6;17:40. 28061850 10.1186/s12889-016-3986-9 PMC5219779

[25] Tadele MM, Dagnaw A, Alamirew D. Food handling practice and associated factors among food handlers in public food establishments of Ethiopia: a systematic review and meta-analysis. BMJ Open. 2022 Mar 15;12(3):e051310. 10.1136/bmjopen-2021-051310 PMC8928274 35292488

[26] Gashaw A, Gebrehiwot E. Study on Milk Hygiene, Quality Control in the Market Chain in Jimma. Adv Dairy Res. 2018 Jan 1;06.

[27] Abdi AM, Amano A, Abrahim A, Getahun M, Ababor S, Kumie A. Food Hygiene Practices and Associated Factors Among Food Handlers Working in Food Establishments in the Bole Sub City, Addis Ababa, Ethiopia. Risk Manag Healthc Policy. 2020 Oct 1; 13:1861–8. 33061719 10.2147/RMHP.S266342 PMC7535140

[28] Borbon NM, Tolentino Melinda. Food Safety Practices of Food Establishments: Basis for Policy Enhancement. 2020 Jan.

[29] FAO, WHO. General principles of food hygiene. Rome, Italy: FAO, WHO; 2023.

[30] Nasrolahei M, Mirshafiee S, Kholdi S, Salehian M, Nasrolahei M. Bacterial assessment of food handlers in Sari City, Mazandaran Province, north of Iran. J Infect Public Health. 2017;10(2):171–6. doi: 10.1016/j.jiph.2016.03.006 27435639

[31] Vicar E, Alo D, Koyiri V, Opare-Asamoah K, Obeng-Bempong M, Mensah G. Carriage of Antibiotic Resistant Bacteria and Associated Factors Among Food Handlers in Tamale Metropolis, Ghana: Implications for Food Safety. Microbiol Insights. 2023 Jan 28; 16:117863612211506. 10.1177/11786361221150695 PMC9885032 36726578

[32] WHO. Five keys to safer food manual [Internet]. 2019 [cited 2023 Sep 8]. Available from: https://www.who.int/publications-detail-redirect/five-keys-to-safer-food-manual

[33] Lema K, Abuhay N, Kindie W, Dagne H, Guadu T. Food Hygiene Practice and Its Determinants Among Food Handlers at University of Gondar, Northwest Ethiopia, 2019. Int J Gen Med. 2020 Nov 13:1129–37. 33235486 10.2147/IJGM.S262767 PMC7679353

[34] Stratev D, Odeyemi OA, Pavlov A, Kyuchukova R, Fatehi F, Bamidele FA. Food safety knowledge and hygiene practices among veterinary medicine students at Trakia University, Bulgaria. J Infect Public Health. 2017 Nov;10(6):778–82. doi: 10.1016/j.jiph.2016.12.001 28188117

[35] Taha S, Osaili TM, Vij M, Vij A, Alhogaraty E, AL-Utaibi G, et al. Measuring management practices impact on hygiene practices of food handlers: The mediating role of commitment and training perception. Food Control. 2021 Dec; 130:108313.

[36] Taha S, Wilkins S, Juusola K, Osaili TM. Food Safety Performance in Food Manufacturing Facilities: The Influence of Management Practices on Food Handler Commitment. J Food Prot. 2020 Jan 1; 83(1):60–7. 31841360 10.4315/0362-028X.JFP-19-126

[37] Mengist A, Mengistu G, Reta A. Prevalence and antimicrobial susceptibility pattern of Salmonella and Shigella among food handlers in catering establishments at Debre Markos University, Northwest Ethiopia. Int J Infect Dis. 2018;75:74–9. doi: 10.1016/j.ijid.2018.08.008 30144555

[38] Ahmednur M, Esmael M, Feresa F. Handwashing Practice of Food Establishment Customers, Microbial Quality of Handwashing Water, and Associated Factors in Ginjo Kebele, Jimma Town, Southwest Ethiopia. Environ Health Insights. 2022 Dec 12; 16:11786302221144197. 36530487 10.1177/11786302221144197 PMC9751165

[39] A M, Y A, A R. Bacterial and Parasitic Assessment from Fingernails in Debre Markos, Northwest Ethiopia. Can J Infect Dis Med Microbiol J Can Mal Infect Microbiol Medicale. 2018 Oct 18. 10.1155/2018/6532014 PMC6220742 30498537

[40] Levine ND, Buchanan RE, Gibbons NE, eds. 1974. Bergey’s Manual of Determinative Bacteriology. 8th ed. Williams & Wilkins Co., Baltimore, Md. 21202. xxvi + 1246 pp. $45.00. J Protozool. 1975;22(1):7–7.

[41] Mama M, Alemu G. Prevalence, antimicrobial susceptibility patterns and associated risk factors of Shigella and Salmonella among food handlers in Arba Minch University, South Ethiopia. BMC Infect Dis. 2016 Nov 21;16(1):686. doi: 10.1186/s12879-016-2035-8 27871241 PMC5117698

[42] Legesse D, Tilahun M, Getehun E, Haftu D. Food Handling Practices and Associated Factors among Food Handlers in Arba Minch Town Public Food Establishments in Gamo Gofa Zone, Southern Ethiopia. Epidemiol Open Access. 2017 Jan 1;07.

[43] Alemayehu T, Aderaw Z, Giza M, Diress G. Food Safety Knowledge, Handling Practices and Associated Factors Among Food Handlers Working in Food Establishments in Debre Markos Town, Northwest Ethiopia, 2020: Institution-Based Cross-Sectional Study. Risk Manag Healthc Policy. 2021 Mar 17;14:1155–63. 33762858 10.2147/RMHP.S295974 PMC7982701

[44] Hamed A, Mohammed N. Food safety knowledge, attitudes and self-reported practices among food handlers in Sohag Governorate, Egypt. East Mediterr Health J Rev Sante Mediterr Orient Al-Majallah Al-Sihhiyah Li-Sharq Al-Mutawassit. 2020 Apr 16;26(4):374–81. 10.26719/emhj.19.047 32338355

[45] Lestantyo D, Husodo AH, Iravati S, Shaluhiyah Z. Safe Food Handling Knowledge, Attitude and Practice of Food Handlers in Hospital Kitchen. Int J Public Health Sci IJPHS. 2017 Dec 1;6(4):324.

[46] Aliyo A, Daba J, Legese M. Sanitary and Hygienic Status and Associated Factors Among Food and Drinking Establishments of Burayu Town, Oromia, Ethiopia. Health Serv Res Manag Epidemiol. 2023 Jan 1; 10:23333928221144553. 36814937 10.1177/23333928221144553 PMC9940228

[47] Ketema S, Areche FO, Alejo OVP, Montesinos CCZ, Rivera TJC, Huaman CWT, et al. Predictors of Food Handling Practices of Massive Food Catering Establishment in Ethiopia: Systematic Review and Meta-Analysis. Curr Res Nutr Food Sci J. 2023 Aug 31;11(2):519–33.

[48] Alamneh AA, Ketema DB, Simieneh MM, Wubie M, Lamore Y, Tessema MT, et al. Food hygiene practice and its associated factors among food handlers working in food establishments during the COVID-19 pandemic in East Gojjam and West Gojjam Zones, North West Ethiopia. SAGE Open Med. 2022 Jan 1;10:20503121221081070. 35223034 10.1177/20503121221081070 PMC8864280

[49] Giné-Garriga R, Delepiere A, Ward R, Alvarez-Sala J, Alvarez-Murillo I, Mariezcurrena V, et al. COVID-19 water, sanitation, and hygiene response: Review of measures and initiatives adopted by governments, regulators, utilities, and other stakeholders in 84 countries. Sci Total Environ. 2021 Nov 15;795:148789. 34243010 10.1016/j.scitotenv.2021.148789 PMC8254381

[50] Vashisht A. Food Safety Culture: An Underlying Cause for Success and Failures of Food Safety Management Systems. In 2018 [cited 2022 Dec 21]. Available from: https://www.semanticscholar.org/paper/Food-Safety-Culture%3A-An-Underlying-Cause-for-and-of-Vashisht/51240291774b9bfa3cb5ceb8c06bc94c834bd888

[51] Chekol FA, Melak MF, Belew AK, Zeleke EG. Food handling practice and associated factors among food handlers in public food establishments, Northwest Ethiopia. BMC Res Notes. 2019;12(1):20. doi: 10.1186/s13104-019-4047-0 30642374 PMC6332519

[52] Yenealem DG, Yallew WW, Abdulmajid S. Food Safety Practice and Associated Factors among Meat Handlers in Gondar Town: A Cross-Sectional Study. J Environ Public Health. 2020 Feb 24; 2020:1–7. 10.1155/2020/7421745 PMC7060434 32184834

[53] Tessema AG, Gelaye KA, Chercos DH. Factors affecting food handling Practices among food handlers of Dangila town food and drink establishments, North West Ethiopia. BMC Public Health. 2014 Jun 7;14(1):571. 24908104 10.1186/1471-2458-14-571 PMC4057591

[54] Azanaw J, Engdaw GT, Dejene H, Bogale S, Degu S. Food hygiene knowledge, and practices and their associated factors of street food vendors in Gondar city, Northwest Ethiopia, 2021: A cross-sectional study. Heliyon. 2022 Nov 1;8(11):e11707. 10.1016/j.heliyon.2022.e11707 PMC9694083 36439770

[55] Nnebue C, ProsperOU Adogu, Ifeadike C, Ironkwe O. Assessment of the food hygiene practices of food handlers in the Federal Capital Territory of Nigeria. Trop J Med Res. 2014;17(1):10.

[56] Fanta F, Azene M, Balcha K, Samson H, Kebede A. Determinants of safe food handling practice among food handlers in food establishments, Yeka sub city, Addis Ababa, Ethiopia. Heliyon. 2023 Jan 12;9: e12977. 10.1016/j.heliyon.2023.e12977 PMC9868874 36699270

[57] Aa L, Is H, Jh D, Jfr L. Bacterial contamination of the hands of food handlers as indicator of hand washing efficacy in some convenient food industries in South Africa. Pak J Med Sci. 2014;30(4):755–8. 25097511 PMC4121692

[58] Allam H, Al-Batanony M, Seif A, Awad E. Hand Contamination among Food Handlers. Br Microbiol Res J. 2016;13(1):1–8.

[59] Havelaar AH, Kirk MD, Torgerson PR, Gibb HJ, Hald T, Lake RJ, et al. World Health Organization Global Estimates and Regional Comparisons of the Burden of Foodborne Disease in 2010. PLoS Med. 2015 Dec;12(12):e1001923. 10.1371/journal.pmed.1001923 PMC4668832 26633896

[60] Tasew H A. Contamination of Bacteria and Associated Factors among Food Handlers Working in the Student Cafeterias of Jimma University Main Campus, Jimma, South West Ethiopia. Altern Integr Med [Internet]. 2015.

[61] Bencardino D, Amagliani G, Brandi G. Carriage of Staphylococcus aureus among food handlers: An ongoing challenge in public health. Food Control. 2021 Dec 1;130:108362.

[62] Assefa T Assessment of Bacterial Hand Contamination and Associated Factors among Food Handlers Working in the Student Cafeterias of Jimma Main Campus, Jimma, South West Ethiopia. J Community Med Health Educ. 2015.

[63] Ekici G, Dümen E. Escherichia coli and Food Safety. In. 2019.

[64] Raposo A, Pérez E, de Faria CT, Ferrús MA, Carrascosa C. Food Spoilage by Pseudomonas spp.—An Overview. In: Foodborne Pathogens and Antibiotic Resistance. John Wiley & Sons, Ltd; 2016.

[65] Kumar H, Franzetti L, Kaushal A, Kumar D. Pseudomonas fluorescens: a potential food spoiler and challenges and advances in its detection. Ann Microbiol. 2019 Sep;69(9):873–83

[66] Awol N, Nigusse D, Ali M. Prevalence and antimicrobial susceptibility profile of Salmonella and Shigella among food handlers working in food establishment at Hawassa city, Southern Ethiopia. BMC Res Notes. 2019;12(1):712. doi: 10.1186/s13104-019-4725-y 31666137 PMC6822460

[67] Akuoko C. Bacterial Reduction of Hand Contamination: Hand Rubbing with Alcohol-Based Solution or Hand Washing with Soap and Water? J Health Med Nurs. 2019 Oct 1.

