# Supplementary material for: Food hygiene practices and associated factors among food handlers in food establishments of Jimma City, Southwest Ethiopia
Source: PLoS One. 2025 May 29;20(5):e0321222. doi: 10.1371/journal.pone.0321222 (PMC12121743; doi:10.1371/journal.pone.0321222)
Supplement: S1 Annex I — (DOCX) [file pone.0321222.s001.docx]

**Annex I: English version of the participant Information Sheet form**

Dear Respondents:

The questionnaire is prepared to evaluate Food Hygiene Practices and Associated Factors Among Food Handlers working in Food Establishments in Jimma City, Southwest Ethiopia. The findings from this study help find solutions to various foodborne diseases, provide basic information for decision-makers, and also provide baseline data for further studies. You are one of the eligible participants for this interview. So, you are kindly requested to answer every question and provide hand swab samples. The data collection procedures might cause minor discomfort and will take 20–30 minutes. The information you provide is confidential and used only for the purpose of this study. If you have any confusion or question, please don’t hesitate to ask the data collector.

Your cooperation and participation until the completion of the questionnaire is very crucial for the successful completion of this assessment.

Thank you in advance for your cooperation!

Questionnaire code: _________

Data collection date: _______________

Data collector’s name and signature: _____________________________ __________

Supervisor’s Name and signature: _______________________________ ___________
