# Supplementary material for: Food hygiene practices and associated factors among food handlers in food establishments of Jimma City, Southwest Ethiopia
Source: PLoS One. 2025 May 29;20(5):e0321222. doi: 10.1371/journal.pone.0321222 (PMC12121743; doi:10.1371/journal.pone.0321222)
Supplement: S2 Annex II — (DOCX) [file pone.0321222.s002.docx]

**Annex II: English Version of the participant Voluntary Consent form and Questionnaire**

I have read the information sheet (or it has been read to me); I have understood that it involves the study of the assessment of food hygiene practices of food handlers working in food establishments in Jimma town. And also, I have clarified the purpose of the study, the procedure to be carried out, the risks associated with the study, the benefits of the study, and the confidentiality of the information. I, the undersigned, confirm that I give consent to participate after a clear understanding of the objectives and conditions of the study and with recognition of my right to withdraw from the study if I change my mind.

I…………………………………………do interestingly give consent to Mr./Mrs./Miss ………………………….to include me in the proposed research. The purpose of the proposal has been explained to me in a language I understand.

| **Part - 01 Socio demographic characteristics of food handler in Jimma City** | | | |
| --- | --- | --- | --- |
| **No** | **Question** | **Response** | **Code** |
| 101 | Sex | 1. Male 2. Female |  |
| 102 | Age | _____ years |  |
| 103 | Your role | 1. Cooker 2. Waiter |  |
| 104 | Type of food establishments you are working in? |  |  |
| 105 | What is your work experience in years? | ___years |  |
| 106 | What is your current marital status? | 1. Single 2. Married 3. Divorced. 4. Widowed |  |
| 107 | What is your educational status? | 1. Can’t read and write 2. Primary education 3. Secondary education 4. Diploma and above |  |
| **Part-02 Food hygiene practices of food handlers in Jimma town** | | | |
| 201 | Do you wear gloves when you handle ready to eat food or prepare food? | 1. Never 2. Rarely 3. Sometimes 4. Most of the times 5. Always |  |
| 202 | Do you work when you have diarrhea? | 1. Never 2. Rarely 3. Sometimes 4. Most of the times 5. Always |  |
| 203 | Do you wash your hand with water and soap after using the toilet? | 1. Never 2. Rarely 3. Sometimes 4. Most of the times 5. Always |  |
| 204 | Do you keep cooked meal (meat) at room temperature for more than 4 h? | 1. Never 2. Rarely 3. Sometimes 4. Most of the times 5. Always |  |
| 205 | Do you work when you have cold? | 1. Never 2. Rarely 3. Sometimes 4. Most of the times 5. Always |  |
| 206 | Do you use water storage equipment in this food establishment for/during water shortage? | 1. Never 2. Rarely 3. Sometimes 4. Most of the times 5. Always |  |
| 207 | Always use clean and separate cutting boards for raw and ready-to-eat food | 1. Never 2. Rarely 3. Sometimes 4. Most of the times 5. Always |  |
| 208 | Do you wear any kind of bracelets during food preparation/serve? | 1. Never 2. Rarely 3. Sometimes 4. Most of the times 5. Always |  |
| **Part-03 Knowledge on food hygiene of food handlers in Jimma town** | | | |
| 301 | Do you know about food borne disease transmission due to contaminated food? | 1. Yes 2. No |  |
| 302 | Do you think improper handling of food could pose health risks to consumers? | 1. Yes 2. No |  |
| 303 | Do you know food prepared in advance reduces the risk of food contamination? | 1. Yes 2. No |  |
| 304 | Do you know germs can be found on cutting board and other food utensils? | 1. Yes 2. No |  |
| 305 | Do you think contaminated foods always have some change in color, odor or taste? | 1. Yes 2. No |  |
| 306 | Do you know health status of workers should be evaluated before employment? | 1. Yes 2. No |  |
| 307 | Do you know use of safe water for cooking purpose can reduces FBDs? | 1. Yes 2. No |  |
| 308 | Do you know vectors (rats and cockroaches) lead for food contamination? | 1. Yes 2. No |  |
| 309 | Do you know hand drying is as important as to hand washing during food handling? | 1. Yes 2. No |  |
| **Part-04 Information on food hygiene attitude of food handlers in Jimma town** | | | |
| 401 | Do you agree improper food storage is dangerous to health? | 1. Strongly disagree 2. Disagree 3. Undecided 4. Agree 5. Strongly agree |  |
| 402 | Do you agree frequent hand-washing during food preparation is worth the extra time? | 1. Strongly disagree 2. Disagree 3. Undecided 4. Agree 5. Strongly agree |  |
| 403 | Do you agree keeping working surfaces and utensils clean reduces the risk of illness? | 1. Strongly disagree 2. Disagree 3. Undecided 4. Agree 5. Strongly agree |  |
| 404 | Do you agree keeping raw and cooked food separate prevent illness? | 1. Strongly disagree 2. Disagree 3. Undecided 4. Agree 5. Strongly agree |  |
| 405 | Do you agree safe food handling is an important part of your job responsibilities? | 1. Strongly disagree 2. Disagree 3. Undecided 4. Agree 5. Strongly agree |  |
| 406 | It is important to throw away foods that have reached their expiry date | 1. Strongly disagree 2. Disagree 3. Undecided 4. Agree 5. Strongly agree |  |
| 407 | Long and painted fingernails could contaminate food with foodborne pathogens | 1. Strongly disagree 2. Disagree 3. Undecided 4. Agree 5. Strongly agree |  |
