# Supplementary material for: Food hygiene practices and associated factors among food handlers in food establishments of Jimma City, Southwest Ethiopia
Source: PLoS One. 2025 May 29;20(5):e0321222. doi: 10.1371/journal.pone.0321222 (PMC12121743; doi:10.1371/journal.pone.0321222)
Supplement: S3 Supplementary Data — (DOCX) [file pone.0321222.s003.docx]

**Datasets of Analyzed Result**

| **Sex of participants** | | | | | |
| --- | --- | --- | --- | --- | --- |
|  | | Frequency | Percent | Valid Percent | Cumulative Percent |
| Valid | Male | 81 | 26.1 | 26.1 | 26.1 |
|  | Female | 229 | 73.9 | 73.9 | 100.0 |
|  | Total | 310 | 100.0 | 100.0 |  |

| **Marital status of Food Handlers** | | | | | |
| --- | --- | --- | --- | --- | --- |
|  | | Frequency | Percent | Valid Percent | Cumulative Percent |
| Valid | Single | 200 | 64.5 | 64.5 | 64.5 |
|  | Married | 98 | 31.6 | 31.6 | 96.1 |
|  | Divorced | 11 | 3.5 | 3.5 | 99.7 |
|  | Widowed | 1 | .3 | .3 | 100.0 |
|  | Total | 310 | 100.0 | 100.0 |  |

| **Age groups of Food Handlers** | | | | | |
| --- | --- | --- | --- | --- | --- |
|  | | Frequency | Percent | Valid Percent | Cumulative Percent |
| Valid | 20 and under | 115 | 37.1 | 37.1 | 37.1 |
|  | 21-30 | 154 | 49.7 | 49.7 | 86.8 |
|  | 31-40 | 34 | 11.0 | 11.0 | 97.7 |
|  | above 40 | 7 | 2.3 | 2.3 | 100.0 |
|  | Total | 310 | 100.0 | 100.0 |  |

| **Educational status** | | | | | |
| --- | --- | --- | --- | --- | --- |
|  | | Frequency | Percent | Valid Percent | Cumulative Percent |
| Valid | Can't read and write | 11 | 3.5 | 3.5 | 3.5 |
|  | Primary Education | 119 | 38.4 | 38.4 | 41.9 |
|  | Secondary Education | 130 | 41.9 | 41.9 | 83.9 |
|  | Diploma and above | 50 | 16.1 | 16.1 | 100.0 |
|  | Total | 310 | 100.0 | 100.0 |  |

| **Experience (Year of Service) of Food Handlers** | | | | | |
| --- | --- | --- | --- | --- | --- |
|  | | Frequency | Percent | Valid Percent | Cumulative Percent |
| Valid | under 2 | 190 | 61.3 | 61.3 | 61.3 |
|  | 2-4 | 80 | 25.8 | 25.8 | 87.1 |
|  | 5-7 | 29 | 9.4 | 9.4 | 96.5 |
|  | 8 and over | 11 | 3.5 | 3.5 | 100.0 |
|  | Total | 310 | 100.0 | 100.0 |  |

| **Role of Participant** | | | | | |
| --- | --- | --- | --- | --- | --- |
|  | | Frequency | Percent | Valid Percent | Cumulative Percent |
| Valid | Cooker | 160 | 51.6 | 51.6 | 51.6 |
|  | Server | 150 | 48.4 | 48.4 | 100.0 |
|  | Total | 310 | 100.0 | 100.0 |  |

| **Type of Food establishments** | | | | | |
| --- | --- | --- | --- | --- | --- |
|  | | Frequency | Percent | Valid Percent | Cumulative Percent |
| Valid | Hotel | 82 | 26.5 | 26.5 | 26.5 |
|  | Bar and Restaurant | 98 | 31.6 | 31.6 | 58.1 |
|  | Cafeteria and Restaurant | 62 | 20.0 | 20.0 | 78.1 |
|  | Snack House | 68 | 21.9 | 21.9 | 100.0 |
|  | Total | 310 | 100.0 | 100.0 |  |

**Knowledge of food Handlers**

|  | | | | | |
| --- | --- | --- | --- | --- | --- |
|  | | Frequency | Percent | Valid Percent | Cumulative Percent |
| **Know about food borne disease transmission due to contaminated food** | No | 119 | 38.4 | 38.4 | 38.4 |
|  | Yes | 191 | 61.6 | 61.6 | 100.0 |
| **Improper handling of food could pose health risks to consumers** | No | 31 | 10.0 | 10.0 | 10.0 |
|  | Yes | 279 | 90.0 | 90.0 | 100.0 |
| **Know food prepared in advance reduces the risk of food contamination** | No | 41 | 13.2 | 13.2 | 13.2 |
|  | Yes | 269 | 86.8 | 86.8 | 100.0 |
| **Know germs can be found on cutting board and other food utensils** | No | 38 | 12.3 | 12.3 | 12.3 |
|  | Yes | 272 | 87.7 | 87.7 | 100.0 |
| **Contaminated foods always have some change in color, odor or taste** | No | 107 | 34.5 | 34.5 | 34.5 |
|  | Yes | 203 | 65.5 | 65.5 | 100.0 |
| **Know health status of workers should be evaluated before employment** | No | 205 | 66.1 | 66.1 | 66.1 |
|  | Yes | 105 | 33.9 | 33.9 | 100.0 |
| **Know use of safe water for cooking purpose can reduces FBDs** | No | 120 | 38.7 | 38.7 | 38.7 |
|  | Yes | 190 | 61.3 | 61.3 | 100.0 |
| **Know vectors (rats and cockroaches) lead for food contamination** | No | 24 | 7.7 | 7.7 | 7.7 |
|  | Yes | 286 | 92.3 | 92.3 | 100.0 |
| **Know hand drying is as important as to hand washing during food handling** | No | 71 | 22.9 | 22.9 | 22.9 |
|  | Yes | 239 | 77.1 | 77.1 | 100.0 |

| **Overall Knowledge of Food Handlers** | | | | | |
| --- | --- | --- | --- | --- | --- |
|  | | Frequency | Percent | Valid Percent | Cumulative Percent |
| Food Handlers’ knowledge | Poor | 211 | 68.1 | 68.1 | 68.1 |
|  | Good | 99 | 31.9 | 31.9 | 100.0 |
|  | Total | 310 | 100.0 | 100.0 |  |

**Attitude of Food Handlers**

|  | | | | | |
| --- | --- | --- | --- | --- | --- |
|  | | Frequency | Percent | Valid Percent | Cumulative Percent |
| **Improper food storage is dangerous to health** | Strongly disagree | 0 | .0 | .0 | .0 |
|  | Disagree | 18 | 5.8 | 5.8 | 5.8 |
|  | Undecided | 62 | 20.0 | 20.0 | 25.8 |
|  | Agree | 180 | 58.1 | 58.1 | 83.9 |
|  | Strongly agree | 50 | 16.1 | 16.1 | 100.0 |
| **Frequent hand-washing during food preparation is worth the extra time** | Strongly disagree | 1 | .3 | .3 | .3 |
|  | Disagree | 28 | 9.0 | 9.0 | 9.4 |
|  | Undecided | 82 | 26.5 | 26.5 | 35.8 |
|  | Agree | 136 | 43.9 | 43.9 | 79.7 |
|  | Strongly agree | 63 | 20.3 | 20.3 | 100.0 |
| **Keeping working surfaces and utensils clean reduces the risk of illness** | Strongly disagree | 1 | .3 | .3 | .3 |
|  | Disagree | 4 | 1.3 | 1.3 | 1.6 |
|  | Undecided | 27 | 8.7 | 8.7 | 10.3 |
|  | Agree | 193 | 62.3 | 62.3 | 72.6 |
|  | Strongly agree | 85 | 27.4 | 27.4 | 100.0 |
| **Keeping raw and cooked food separately could prevent illness** | Strongly disagree | 1 | .3 | .3 | .3 |
|  | Disagree | 20 | 6.5 | 6.5 | 6.8 |
|  | Undecided | 41 | 13.2 | 13.2 | 20.0 |
|  | Agree | 159 | 51.3 | 51.3 | 71.3 |
|  | Strongly agree | 89 | 28.7 | 28.7 | 100.0 |
| **Safe food handling is an important part of your job responsibilities** | Strongly disagree | 2 | .6 | .6 | .6 |
|  | Disagree | 20 | 6.5 | 6.5 | 7.1 |
|  | Undecided | 72 | 23.2 | 23.2 | 30.3 |
|  | Agree | 152 | 49.0 | 49.0 | 79.4 |
|  | Strongly agree | 64 | 20.6 | 20.6 | 100.0 |
| **Important to throw away foods that have reached their expiry date** | Strongly disagree | 1 | .3 | .3 | .3 |
|  | Disagree | 15 | 4.8 | 4.8 | 5.2 |
|  | Undecided | 28 | 9.0 | 9.0 | 14.2 |
|  | Agree | 129 | 41.6 | 41.6 | 55.8 |
|  | Strongly agree | 137 | 44.2 | 44.2 | 100.0 |
| **Long fingernails could contaminate food with foodborne pathogens** | Strongly disagree | 1 | .3 | .3 | .3 |
|  | Disagree | 48 | 15.5 | 15.5 | 15.8 |
|  | Undecided | 54 | 17.4 | 17.4 | 33.2 |
|  | Agree | 118 | 38.1 | 38.1 | 71.3 |
|  | Strongly agree | 89 | 28.7 | 28.7 | 100.0 |

| **Overall Attitude of Food handlers** | | | | | |
| --- | --- | --- | --- | --- | --- |
|  | | Frequency | Percent | Valid Percent | Cumulative Percent |
| Food handlers’ Attitude | Negative | 206 | 66.5 | 66.5 | 66.5 |
|  | Positive | 104 | 33.5 | 33.5 | 100.0 |
|  | Total | 310 | 100.0 | 100.0 |  |

**Food handlers Food Hygienic Practice**

|  | | | | | | |
| --- | --- | --- | --- | --- | --- | --- |
|  | | Frequency | Percent | Valid Percent | | Cumulative Percent |
| **use clean and separate cutting boards for raw and ready-to-eat food** | Never | 0 | .0 | | .0 | .0 |
|  | Rarely | 23 | 7.4 | | 7.4 | 7.4 |
|  | Sometimes | 19 | 6.1 | | 6.1 | 13.5 |
|  | Most of the times | 126 | 40.6 | | 40.6 | 54.2 |
|  | Always | 142 | 45.8 | | 45.8 | 100.0 |
| **use water storage equipment in this food establishment** | Never | 4 | 1.3 | | 1.3 | 1.3 |
|  | Rarely | 18 | 5.8 | | 5.8 | 7.1 |
|  | Sometimes | 46 | 14.8 | | 14.8 | 21.9 |
|  | Most of the times | 136 | 43.9 | | 43.9 | 65.8 |
|  | Always | 106 | 34.2 | | 34.2 | 100.0 |
| **Always wash your hand with water and soap after using the restroom/toilet** | Never | 3 | 1.0 | | 1.0 | 1.0 |
|  | Rarely | 5 | 1.6 | | 1.6 | 2.6 |
|  | Sometimes | 24 | 7.7 | | 7.7 | 10.3 |
|  | Most of the times | 181 | 58.4 | | 58.4 | 68.7 |
|  | Always | 97 | 31.3 | | 31.3 | 100.0 |
| **Wear gloves when you handle ready to eat food** | Never | 9 | 2.9 | | 2.9 | 2.9 |
|  | Rarely | 83 | 26.8 | | 26.8 | 29.7 |
|  | Sometimes | 112 | 36.1 | | 36.1 | 65.8 |
|  | Most of the times | 94 | 30.3 | | 30.3 | 96.1 |
|  | Always | 12 | 3.9 | | 3.9 | 100.0 |
| **I don’t wear any kind of bracelets during food serve/preparation** | Never | 6 | 1.9 | | 1.9 | 1.9 |
|  | Rarely | 76 | 24.5 | | 24.5 | 26.5 |
|  | Sometimes | 58 | 18.7 | | 18.7 | 45.2 |
|  | Most of the times | 137 | 44.2 | | 44.2 | 89.4 |
|  | Always | 33 | 10.6 | | 10.6 | 100.0 |
| **I don’t work when I have diarrhea** | Never | 6 | 1.9 | | 1.9 | 1.9 |
|  | Rarely | 101 | 32.6 | | 32.6 | 34.5 |
|  | Sometimes | 97 | 31.3 | | 31.3 | 65.8 |
|  | Most of the times | 102 | 32.9 | | 32.9 | 98.7 |
|  | Always | 4 | 1.3 | | 1.3 | 100.0 |
| **I don’t keep cooked meal at room temperature for more than 4 hours** | Never | 6 | 1.9 | | 1.9 | 1.9 |
|  | Rarely | 59 | 19.0 | | 19.0 | 21.0 |
|  | Sometimes | 74 | 23.9 | | 23.9 | 44.8 |
|  | Most of the times | 152 | 49.0 | | 49.0 | 93.9 |
|  | Always | 19 | 6.1 | | 6.1 | 100.0 |
| **I don’t work when I have cold** | Never | 14 | 4.5 | | 4.5 | 4.5 |
|  | Rarely | 186 | 60.0 | | 60.0 | 64.5 |
|  | Sometimes | 69 | 22.3 | | 22.3 | 86.8 |
|  | Most of the times | 38 | 12.3 | | 12.3 | 99.0 |
|  | Always | 3 | 1.0 | | 1.0 | 100.0 |

| **Overall Food Handlers’ Food Hygiene practice status** | | | | | |
| --- | --- | --- | --- | --- | --- |
|  | | Frequency | Percent | Valid Percent | Cumulative Percent |
| Food hygiene practice level | Poor | 222 | 71.6 | 71.6 | 71.6 |
|  | Good | 88 | 28.4 | 28.4 | 100.0 |
|  | Total | 310 | 100.0 | 100.0 |  |

| **Presence of isolated bacteria on Hands of Food handlers** | | | | | |
| --- | --- | --- | --- | --- | --- |
|  | | Frequency | Percent | Valid Percent | Cumulative Percent |
| Presence of Isolated bacteria | Yes | 140 | 45.2 | 45.2 | 45.2 |
|  | No | 170 | 54.8 | 54.8 | 100.0 |
|  | Total | 310 | 100.0 | 100.0 |  |

| **Frequencies of Isolated bacteria** | | | | |
| --- | --- | --- | --- | --- |
|  | | Responses | | Percent of Cases |
|  |  | N | Percent |  |
| Type of Isolated b | S. aureus | 105 | 46.9% | 75.0% |
|  | E. coli | 31 | 13.8% | 22.1% |
|  | Pseudomonas | 43 | 19.2% | 30.7% |
|  | Salmonella | 21 | 9.4% | 15.0% |
|  | Shigella | 24 | 10.7% | 17.1% |
| Total | | 224 | 100.0% | 160.0% |
|  | | | | |

**Presence of Isolated bacteria with respect to Food Handlers practice**

| **Chi-Square Tests** | | | | | | | |  |  |  |  |
| --- | --- | --- | --- | --- | --- | --- | --- | --- | --- | --- | --- |
|  | | Value | | df | Asymptotic Significance (2-sided) | | |  |  |  |  |
| Spearman correlation | | 68.465 | | 3 | .000 | | |  |  |  |  |
| Likelihood Ratio | | 73.945 | | 3 | .000 | | |  |  |  |  |
| Linear-by-Linear Association | | 52.630 | | 1 | .000 | | |  |  |  |  |
| N of Valid Cases | | 310 | |  |  | | |  |  |  |  |
|  | | | | | | | |  |  |  |  |
| Presence of isolated bacteria * Experience of participants (group 1) | | | | | | | |  |  |  |  |
|  | | | | | | Experience in group1 | | | | | Total |
|  |  |  |  |  |  | under 2 | 2-4 | | 5-7 | 8 and over |  |
| Presence of isolated bacteria | Yes | | Count | | | 121 | 14 | | 3 | 2 | 140 |
|  |  |  | % within Presence of isolated bacteria | | | 86.4% | 10.0% | | 2.1% | 1.4% | 100.0% |
|  |  |  | % within Experience in group1 | | | 63.7% | 17.5% | | 10.3% | 18.2% | 45.2% |
|  | No | | Count | | | 69 | 66 | | 26 | 9 | 170 |
|  |  |  | % within Presence of isolated bacteria | | | 40.6% | 38.8% | | 15.3% | 5.3% | 100.0% |
|  |  |  | % within Experience in group1 | | | 36.3% | 82.5% | | 89.7% | 81.8% | 54.8% |
| Total | | | Count | | | 190 | 80 | | 29 | 11 | 310 |
|  |  |  | % within Presence of isolated bacteria | | | 61.3% | 25.8% | | 9.4% | 3.5% | 100.0% |
|  |  |  | % within Experience in group1 | | | 100.0% | 100.0% | | 100.0% | 100.0% | 100.0% |

**Final multivariable logistics regression (Backward LR)**

| **Hosmer and Lemeshow Test** | | | |
| --- | --- | --- | --- |
| Step | Chi-square | df | Sig. |
| 1 | 11.182 | 8 | .192 |

| **Model Summary** | | | |
| --- | --- | --- | --- |
| Step | -2 Log likelihood | Cox & Snell R Square | Nagelkerke R Square |
| 1 | 215.855^a^ | .388 | .557 |
| a. Estimation terminated at iteration number 20 because maximum iterations has been reached. Final solution cannot be found. | | | |

| **Variables in the Equation** | | | | | | | | | | | | | | | | | | |
| --- | --- | --- | --- | --- | --- | --- | --- | --- | --- | --- | --- | --- | --- | --- | --- | --- | --- | --- |
|  | | | | | | | | | | | | | | | | | | |
|  | | | | B | S.E. | | Wald | | df | | Sig. | | Exp(B) | | 95% C.I. for EXP(B) | | | |
|  |  |  |  |  |  |  |  |  |  |  |  |  |  |  | Lower | | Upper | |
| Step 1^a^ | Knowledge group 2(1) | | 2.089 | | .401 | 27.140 | | 1 | | .000 | | 8.080 | | 3.682 | | 17.734 | |  |
|  | Age (1) | | .579 | | .445 | 1.692 | | 1 | | .193 | | 1.785 | | .746 | | 4.273 | |  |
|  | Attitude status 2(1) | | .708 | | .386 | 3.367 | | 1 | | .067 | | 2.029 | | .953 | | 4.321 | |  |
|  | Sex of participant (1) | | .631 | | .442 | 2.038 | | 1 | | .153 | | 1.879 | | .790 | | 4.468 | |  |
|  | Type of Food establishment | |  | |  | 14.684 | | 3 | | .002 | |  | |  | |  | |  |
|  | Type of Food establishment (1) | | -1.139 | | recode.471 | 5.832 | | 1 | | .016 | | .320 | | .127 | | .807 | |  |
|  | Type of Food establishment (2) | | -1.170 | | .498 | 5.525 | | 1 | | .019 | | .310 | | .117 | | .823 | |  |
|  | Type of Food establishment (3) | | -2.015 | | .544 | 13.733 | | 1 | | .000 | | .133 | | .046 | | .387 | |  |
|  | Role of Participant (1) | | -.378 | | .414 | .830 | | 1 | | .362 | | .686 | | .304 | | 1.545 | |  |
|  | Experience in group1 | |  | |  | 3.094 | | 3 | | .377 | |  | |  | |  | |  |
|  | Experience in group1(1) | | .708 | | .423 | 2.801 | | 1 | | .094 | | 2.029 | | .886 | | 4.649 | |  |
|  | Experience in group1(2) | | .732 | | .640 | 1.312 | | 1 | | .252 | | 2.080 | | .594 | | 7.286 | |  |
|  | Experience in group1(3) | | .365 | | 1.052 | .120 | | 1 | | .729 | | 1.441 | | .183 | | 11.326 | |  |
|  | Presence of isolated bacteria (1) | | .737 | | .421 | 3.057 | | 1 | | .080 | | 2.089 | | .915 | | 4.770 | |  |
|  | Educational status | |  | |  | .026 | | 3 | | .999 | |  | |  | |  | |  |
|  | Educational status (1) | | 18.646 | | 11518.849 | .000 | | 1 | | .999 | | 125284456.233 | | .000 | | . | |  |
|  | Educational status (2) | | 18.580 | | 11518.849 | .000 | | 1 | | .999 | | 117261708.185 | | .000 | | . | |  |
|  | Educational status (3) | | 18.617 | | 11518.849 | .000 | | 1 | | .999 | | 121668695.316 | | .000 | | . | |  |
|  | Marital status | |  | |  | 5.981 | | 3 | | .113 | |  | |  | |  | |  |
|  | Marital status (1) | | .162 | | .438 | .137 | | 1 | | .711 | | 1.176 | | .499 | | 2.773 | |  |
|  | Marital status (2) | | -2.268 | | 1.027 | 4.875 | | 1 | | .027 | | .103 | | .014 | | .775 | |  |
|  | Marital status (3) | | -20.910 | | 40192.970 | .000 | | 1 | | 1.000 | | .000 | | .000 | | . | |  |
|  | Constant | | -21.162 | | 11518.849 | .000 | | 1 | | .999 | | .000 | |  | |  | |  |
| Step 2^a^ | | Knowledge group 2(1) | | 2.122 | .375 | | 32.014 | | 1 | | .000 | | 8.344 | | 4.001 | | 17.399 | |
|  |  | Age recode (1) | | .586 | .445 | | 1.729 | | 1 | | .189 | | 1.796 | | .750 | | 4.300 | |
|  |  | Attitude status 2(1) | | .745 | .376 | | 3.920 | | 1 | | .048 | | 2.106 | | 1.007 | | 4.403 | |
|  |  | Sex of participant (1) | | .612 | .433 | | 1.999 | | 1 | | .157 | | 1.845 | | .789 | | 4.312 | |
|  |  | Type of Food establishment | |  |  | | 15.158 | | 3 | | .002 | |  | |  | |  | |
|  |  | Type of Food establishment (1) | | -1.140 | .466 | | 5.980 | | 1 | | .014 | | .320 | | .128 | | .798 | |
|  |  | Type of Food establishment (2) | | -1.162 | .492 | | 5.566 | | 1 | | .018 | | .313 | | .119 | | .821 | |
|  |  | Type of Food establishment (3) | | -2.035 | .541 | | 14.169 | | 1 | | .000 | | .131 | | .045 | | .377 | |
|  |  | Role of Participant (1) | | -.410 | .411 | | .994 | | 1 | | .319 | | .663 | | .296 | | 1.486 | |
|  |  | Experience in group1 | |  |  | | 3.150 | | 3 | | .369 | |  | |  | |  | |
|  |  | Experience in group1(1) | | .713 | .421 | | 2.860 | | 1 | | .091 | | 2.039 | | .893 | | 4.657 | |
|  |  | Experience in group1(2) | | .720 | .635 | | 1.288 | | 1 | | .256 | | 2.055 | | .592 | | 7.127 | |
|  |  | Experience in group1(3) | | .298 | 1.024 | | .085 | | 1 | | .771 | | 1.347 | | .181 | | 10.021 | |
|  |  | Presence of isolated bacteria (1) | | .748 | .419 | | 3.187 | | 1 | | .074 | | 2.114 | | .929 | | 4.807 | |
|  |  | Marital status | |  |  | | 6.298 | | 3 | | .098 | |  | |  | |  | |
|  |  | Marital status (1) | | .148 | .433 | | .117 | | 1 | | .733 | | 1.160 | | .496 | | 2.710 | |
|  |  | Marital status (2) | | -2.323 | 1.013 | | 5.257 | | 1 | | .022 | | .098 | | .013 | | .714 | |
|  |  | Marital status (3) | | -20.886 | 40192.970 | | .000 | | 1 | | 1.000 | | .000 | | .000 | | . | |
|  |  | Constant | | -2.581 | .693 | | 13.856 | | 1 | | .000 | | .076 | |  | |  | |
| Step 3^a^ | | Knowledge group 2(1) | | 2.131 | .370 | | 33.104 | | 1 | | .000 | | 8.426 | | 4.077 | | 17.415 | |
|  |  | Age recode (1) | | .672 | .436 | | 2.378 | | 1 | | .123 | | 1.958 | | .834 | | 4.597 | |
|  |  | Attitude status 2(1) | | .789 | .370 | | 4.560 | | 1 | | .033 | | 2.202 | | 1.067 | | 4.543 | |
|  |  | Sex of participant (1) | | .554 | .421 | | 1.728 | | 1 | | .189 | | 1.740 | | .762 | | 3.974 | |
|  |  | Type of Food establishment | |  |  | | 14.981 | | 3 | | .002 | |  | |  | |  | |
|  |  | Type of Food establishment (1) | | -1.092 | .457 | | 5.699 | | 1 | | .017 | | .336 | | .137 | | .822 | |
|  |  | Type of Food establishment (2) | | -1.114 | .485 | | 5.273 | | 1 | | .022 | | .328 | | .127 | | .849 | |
|  |  | Type of Food establishment (3) | | -2.009 | .535 | | 14.086 | | 1 | | .000 | | .134 | | .047 | | .383 | |
|  |  | Role of Participant (1) | | -.592 | .390 | | 2.301 | | 1 | | .129 | | .553 | | .257 | | 1.189 | |
|  |  | Presence of isolated bacteria (1) | | 1.059 | .381 | | 7.719 | | 1 | | .005 | | 2.884 | | 1.366 | | 6.089 | |
|  |  | Marital status | |  |  | | 6.700 | | 3 | | .082 | |  | |  | |  | |
|  |  | Marital status (1) | | .173 | .431 | | .161 | | 1 | | .689 | | 1.189 | | .510 | | 2.769 | |
|  |  | Marital status (2) | | -2.303 | .989 | | 5.421 | | 1 | | .020 | | .100 | | .014 | | .695 | |
|  |  | Marital status (3) | | -20.723 | 40192.970 | | .000 | | 1 | | 1.000 | | .000 | | .000 | | . | |
|  |  | Constant | | -2.462 | .669 | | 13.558 | | 1 | | .000 | | .085 | |  | |  | |
| Step 4^a^ | | Knowledge group 2(1) | | 2.117 | .368 | | 33.090 | | 1 | | .000 | | 8.306 | | 4.038 | | 17.085 | |
|  |  | Age recode (1) | | .628 | .431 | | 2.120 | | 1 | | .145 | | 1.873 | | .805 | | 4.361 | |
|  |  | Attitude status 2(1) | | .790 | .367 | | 4.630 | | 1 | | .031 | | 2.203 | | 1.073 | | 4.524 | |
|  |  | Type of Food establishment | |  |  | | 13.919 | | 3 | | .003 | |  | |  | |  | |
|  |  | Type of Food establishment (1) | | -1.000 | .447 | | 5.008 | | 1 | | .025 | | .368 | | .153 | | .883 | |
|  |  | Type of Food establishment (2) | | -1.032 | .478 | | 4.660 | | 1 | | .031 | | .356 | | .140 | | .909 | |
|  |  | Type of Food establishment (3) | | -1.875 | .520 | | 12.982 | | 1 | | .000 | | .153 | | .055 | | .425 | |
|  |  | Role of Participant (1) | | -.747 | .373 | | 4.004 | | 1 | | .045 | | .474 | | .228 | | .985 | |
|  |  | Presence of isolated bacteria (1) | | 1.020 | .376 | | 7.370 | | 1 | | .007 | | 2.773 | | 1.328 | | 5.789 | |
|  |  | Marital status | |  |  | | 6.997 | | 3 | | .072 | |  | |  | |  | |
|  |  | Marital status (1) | | .214 | .428 | | .249 | | 1 | | .618 | | 1.238 | | .535 | | 2.866 | |
|  |  | Marital status (2) | | -2.294 | .983 | | 5.449 | | 1 | | .020 | | .101 | | .015 | | .692 | |
|  |  | Marital status (3) | | -20.627 | 40192.970 | | .000 | | 1 | | 1.000 | | .000 | | .000 | | . | |
|  |  | Constant | | -2.014 | .558 | | 13.023 | | 1 | | .000 | | .133 | |  | |  | |
| Step 5^a^ | | Knowledge group 2(1) | | 2.106 | .364 | | 33.466 | | 1 | | .000 | | 8.219 | | 4.026 | | 16.778 | |
|  |  | Attitude status 2(1) | | .842 | .363 | | 5.377 | | 1 | | .020 | | 2.321 | | 1.139 | | 4.730 | |
|  |  | Type of Food establishment | |  |  | | 13.630 | | 3 | | .003 | |  | |  | |  | |
|  |  | Type of Food establishment (1) | | -1.005 | .448 | | 5.031 | | 1 | | .025 | | .366 | | .152 | | .881 | |
|  |  | Type of Food establishment (2) | | -1.053 | .474 | | 4.926 | | 1 | | .026 | | .349 | | .138 | | .884 | |
|  |  | Type of Food establishment (3) | | -1.848 | .520 | | 12.647 | | 1 | | .000 | | .158 | | .057 | | .436 | |
|  |  | Role of Participant (1) | | -.740 | .372 | | 3.960 | | 1 | | .047 | | .477 | | .230 | | .989 | |
|  |  | Presence of isolated bacteria (1) | | 1.082 | .373 | | 8.408 | | 1 | | .004 | | 2.952 | | 1.420 | | 6.134 | |
|  |  | Marital status | |  |  | | 7.460 | | 3 | | .059 | |  | |  | |  | |
|  |  | Marital status (1) | | .465 | .391 | | 1.410 | | 1 | | .235 | | 1.592 | | .739 | | 3.428 | |
|  |  | Marital status (2) | | -2.026 | .964 | | 4.420 | | 1 | | .036 | | .132 | | .020 | | .872 | |
|  |  | Marital status (3) | | -20.383 | 40192.970 | | .000 | | 1 | | 1.000 | | .000 | | .000 | | . | |
|  |  | Constant | | -1.738 | .521 | | 11.155 | | 1 | | .001 | | .176 | |  | |  | |
| 1. Variable(s) entered on step 1: Knowledge group 2, Age recode, Attitude status 2, Sex of participant, Type of Food establishment, Role of Participant, Experience in group1, Presence of isolated bacteria, Educational status, Marital status. | | | | | | | | | | | | | | | | | | |

**Multicollinearity Issue**

| **Coefficients^’^** | | | |
| --- | --- | --- | --- |
| Model | | Collinearity Statistics | |
|  |  | Tolerance | VIF |
| 1 | Sex of participant | .700 | 1.428 |
|  | Type of Food establishment | .642 | 1.559 |
|  | Role of Participant | .671 | 1.490 |
|  | Marital status | .534 | 1.873 |
|  | Educational status | .587 | 1.703 |
|  | Wear gloves when you handle ready to eat food | .583 | 1.714 |
|  | Work when you have diarrhea | .606 | 1.650 |
|  | Always wash your hand with water and soap after using the bath/restroom | .620 | 1.612 |
|  | keep cooked meal (meat) at room temperature for more than 4 h | .646 | 1.548 |
|  | work when you have cold | .560 | 1.785 |
|  | use water storage equipment in this food establishment | .673 | 1.486 |
|  | use clean and separate cutting boards for raw and ready-to-eat food | .668 | 1.497 |
|  | Wear any kind of bracelets during food preparation/serve | .566 | 1.768 |
|  | Know about food borne disease transmission due to contaminated food | .566 | 1.767 |
|  | Improper handling of food could pose health risks to consumers | .758 | 1.319 |
|  | Know food prepared in advance reduces the risk of food contamination | .662 | 1.510 |
|  | Know germs can be found on cutting board and other food utensils | .737 | 1.357 |
|  | Contaminated foods always have some change in color, odor or taste | .582 | 1.718 |
|  | Know health status of workers should be evaluated before employment | .468 | 2.138 |
|  | Know use of safe water for cooking purpose can reduces FBDs | .412 | 2.424 |
|  | Know vectors (rats and cockroaches) lead for food contamination | .785 | 1.274 |
|  | Know hand drying is as important as to hand washing during food handling | .612 | 1.633 |
|  | Improper food storage is dangerous to health | .589 | 1.696 |
|  | Frequent hand-washing during food preparation is worth the extra time | .470 | 2.127 |
|  | Keeping working surfaces and utensils clean reduces the risk of illness | .496 | 2.018 |
|  | Keeping raw and cooked food separate prevent illness | .519 | 1.926 |
|  | Safe food handling is an important part of your job responsibilities | .565 | 1.770 |
|  | Important to throw away foods that have reached their expiry date | .582 | 1.718 |
|  | Long and painted fingernails could contaminate food with foodborne pathogens | .620 | 1.613 |
|  | Presence of isolated bacteria | .603 | 1.659 |
|  | Age groups of FHs | .509 | 1.963 |
|  | Experience of FHs | .610 | 1.639 |
|  | Attitude of FHs | .227 | 4.406 |
|  | Knowledge of FHs | .232 | 4.312 |
| a. Dependent Variable: Hygiene practice status; FHs: Food handlers | | | |

| **Residuals Statistics** | | | | | |
| --- | --- | --- | --- | --- | --- |
|  | Minimum | Maximum | Mean | Std. Deviation | N |
| Predicted Value | .52 | 2.39 | 1.28 | .381 | 310 |
| Std. Predicted Value | -2.014 | 2.893 | .000 | 1.000 | 310 |
| Standard Error of Predicted Value | .082 | .172 | .112 | .014 | 310 |
| Adjusted Predicted Value | .41 | 2.46 | 1.28 | .387 | 310 |
| Residual | -.521 | .653 | .000 | .243 | 310 |
| Std. Residual | -1.951 | 2.447 | .000 | .908 | 310 |
| Stud. Residual | -2.129 | 2.684 | -.001 | 1.003 | 310 |
| Deleted Residual | -.653 | .786 | -.001 | .296 | 310 |
| Stud. Deleted Residual | -2.144 | 2.717 | -.001 | 1.006 | 310 |
| Mahal. Distance | 28.163 | 127.148 | 53.826 | 13.471 | 310 |
| Cook's Distance | .000 | .027 | .004 | .005 | 310 |
| Centered Leverage Value | .091 | .411 | .174 | .044 | 310 |
| a. Dependent Variable: Hygiene practice status | | | | | |

Mahal distance since 127 greater than 52 accepted, which is 53-1

Cook’s distance should be 0-1 which is also accepted
